# Decoding innate lymphoid cell heterogeneity and plasticity in colorectal cancer

**DOI:** 10.1002/ctm2.70593

**Published:** 2026-01-13

**Authors:** Shuomin Zhang, Qingfeng Fu, Zhengyang Xu, Sijun Wang, Guoju You, Xiaoyu Su, Xiaotong Yuan, Chao Liu, Chen Liu, Chaojun Zhang, Bing Liu, Yandong Gong

**Affiliations:** ^1^ Medical School of Chinese PLA Beijing China; ^2^ Department of General Surgery The First Medical Center of Chinese PLA General Hospital Beijing China; ^3^ State Key Laboratory of Experimental Hematology Haihe Laboratory of Cell Ecosystem Senior Department of Hematology Fifth Medical Center Chinese PLA General Hospital Beijing China; ^4^ Post‐Doctoral Scientific Research Station of Basic Medicine Jinan University Guangzhou China; ^5^ State Key Laboratory of Experimental Hematology National Clinical Research Center for Blood Diseases Haihe Laboratory of Cell Ecosystem Institute of Hematology and Blood Diseases Hospital Chinese Academy of Medical Sciences and Peking Union Medical College Tianjin China; ^6^ Laboratory Center Affiliated People's Hospital of Jiangsu University Zhenjiang China; ^7^ Department of Radiation Oncology Peking University First Hospital Beijing China

**Keywords:** colorectal cancer, innate lymphoid cells, single‐cell RNA sequencing, spatial transcriptomics, tumour microenvironment

## Abstract

**Background:**

In colorectal cancer (CRC), innate lymphoid cells (ILCs) play a vital role in preserving and modulating immune homeostasis within the intestinal environment. However, the origins and diverse functions of ILCs in CRC remain poorly understood, making it difficult to clarify how these cells contribute to disease progression and influence therapeutic efficacy.

**Methods:**

Single‐cell RNA sequencing (scRNA‐seq) generated an atlas of ILCs from multiple tissues (bone marrow, blood, and intestine), revealing their origins, heterogeneity, and plasticity. Spatial transcriptomics (ST) and immunofluorescence (IF) defined their specific cellular neighbourhoods within the tumour microenvironment.  In *vitro* co‐culture assays were performed to validate the regulatory role of ILC2s in B cell maturation. Bulk RNA sequencing and flow cytometry were employed to assess the survival and therapeutic response potential of ILCs.

**Results:**

Intestinal ILCs have two distinct origins: ILC3‐CD83 cells derived from the fetal gut, which persist into adulthood; and ILC2 and ILC3‐S100A4 cells that might originate from the bone marrow and migrate through the circulation to colonise intestinal tissues. The tissue‐resident ILC3 subsets exhibited diverse functional roles in CRC. Specifically, trajectory analysis showed that ILC3s differentiated into either stress‐responsive ILC3‐HSPA1B cells or cytotoxic ILC1/NK cells in CRC. Additionally, by using spatial transcriptomics analysis combined with functional assays, we found that bone marrow‐derived ILC2s preferentially localise in tertiary lymphoid structures (TLSs), where they likely support B cell maturation. Notably, higher ILC2 abundance correlated with better clinical outcomes and greater therapeutic benefit.

**Conclusions:**

This study reveals the distinct origins and functional heterogeneity of intestinal ILC subsets in CRC. The enrichment of bone marrow‐derived ILC2s in TLSs, where they likely support B cell maturation, is associated with improved prognosis and favourable immunotherapy response, which may serve as biomarkers for survival and therapeutic efficacy in CRC.

## Graphical abstract

This schematic outlines the origin, heterogeneity, and plasticity of ILCs in colorectal cancer. Created in BioRender. Yiyi, X. (2026) https://BioRender.com/0kwrvq7.

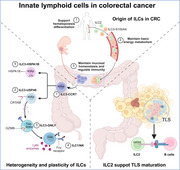

## 1 INTRODUCTION

Globally, colorectal cancer (CRC) represents a significant public health burden, standing as the third most frequently diagnosed malignancy and the second leading cause of cancer‐related death.^1^, ^2^, ^3^, ^4^ Although surgical intervention continues to be the fundamental strategy for curative intent, effective therapeutic options for advanced‐stage CRC patients remain limited, largely owing to the tumour's marked heterogeneity and biological complexity.^5^, ^6^, ^7^ Nevertheless, the advent of immunotherapy has redefined the treatment paradigm in oncology, with durable clinical benefit in several malignancies. Despite the remarkable therapeutic efficacy of PD‐1 and PD‐L1 blockade strategies in a wide array of other malignancies,^8^, ^9^, ^10^, ^11^ their general clinical utility within the context of CRC has proven limited. This suboptimal performance is principally ascribed to the multifaceted and inhibitory nature of the tumour microenvironment, which significantly curtails the establishment of long‐lasting anti‐tumour immune responses. Consequently, the urgent need to overcome these barriers has ignited substantial research into immune effector lineages other than traditional T lymphocytes, identifying them as promising candidates for novel therapeutic interventions.^12^, ^13^

Innate lymphoid cells (ILCs) comprise a diverse group of innate immune cells defined by their lack of antigen receptor rearrangements. They are predominantly localised within mucosal barriers, such as the lung, skin, and gastrointestinal tract.^14^, ^15^, ^16^, ^17^, ^18^ Based on their unique signatures of cytokine release and transcription factor expression, ILCs are classified into five major subsets: natural killer (NK) cells, ILC1s, ILC2s, ILC3s, and lymphoid tissue inducer (LTi) cells.^14^, ^16^, ^19^, ^20^ Under physiological conditions, ILCs orchestrate key processes including lymphoid organogenesis, epithelial barrier maintenance, and rapid immune defence against microbial invasion.^21^, ^22^ Their functional and phenotypic diversity across various tissues and microenvironments highlights their central roles in immune homeostasis.^20^, ^23^, ^24^ Increasingly, investigators have highlighted the capacity of ILCs to modulate critical physiological and pathological states, including inflammation, immunological tolerance, and tumourigenesis. However, their functions are context‐dependent, shaped by factors such as adaptive immunity and disease progression. In addition, ILCs exhibit remarkable plasticity, which allows for context‐dependent remodelling and fate transitions, particularly in response to inflammatory signals or tumours.^25^ For instance, ILC3s can acquire ILC1‐like characteristics in the inflamed intestine, a process that involves the loss of RORγt expression and a shift towards an ILC1‐like state. This transition is catalysed by IL‐12 and IL‐15, which promote T‐bet expression and type 1 cytokine secretion while simultaneously suppressing RORγt.^26^, ^27^, ^28^, ^29^ Notably, this alteration is reversible. In vitro stimulation with IL‐1β, IL‐23, and retinoic acid has been shown to restore the original phenotype.^30^ Such functional flexibility enables rapid cellular adaptation to fluctuating environmental conditions, particularly during infectious or inflammatory episodes.

Although mature helper ILCs were characterised as tissue‐resident populations that expand locally under homeostatic conditions, mouse studies tracking ILC2s have challenged this paradigm by demonstrating context‐dependent egress and reseeding of distant tissues.^31^ Like other immune cells, ILCs express diverse chemokine receptors, integrins and selectins, enabling migration within and between organs and directed trafficking to inflamed sites where they mediate their functions.^32^, ^33^, ^34^, ^35^, ^36^ Recent lineage‐tracing studies further revealed that fetal liver‐derived PD‐1^+^ ILC precursors (ILCPs) migrated to peripheral tissues during embryogenesis, where they locally differentiated into ILC1/3Ps or ILC2Ps, persisted postnatally and sustained tissue ILC pools independent of adult marrow output. In contrast, adult bone marrow primarily supports the generation of ILC2s.^37^

Accumulating evidence indicates that ILCs materially shape colorectal cancer biology. NK cells are central to early anti‐tumour surveillance, and higher intratumoural NK cell density has been reported to be associated with more favourable clinical outcomes. Nevertheless, in CRC, these cells frequently exhibit diminished cytotoxicity and altered receptor programs, and redirect towards an ILC1‐like state, influenced by TGF‐β in the tumour microenvironment. Such a transformation from NK to ILC1 usually indicates a poor prognosis and resistance to treatment.^38^, ^39^, ^40^ Beyond NK cells, other ILC subsets contribute in context‐dependent manners. ILC3s, driven by IL‐23, can sustain colon tumourigenesis through IL‐22‐dependent STAT3 activation in epithelial cells. Recent work further implicates the IL22‐oncostatin M axis amplifies pro‐tumour signalling in the inflamed colon.^41^, ^42^ In contrast, ILC2s have been proven to have a bidirectional effect. IL‐33 can stimulate ILC2s to produce IL‐13, thereby promoting the formation of myeloid suppressor cells derived from monocytes and facilitating immune evasion. On the other hand, emerging evidence highlights a protective capacity within this lineage. Specifically, the secretion of IL‐9 by ILC2s has been observed to potentiate CD8^+^ T cell responses, thereby inhibiting the progression of colorectal neoplasms.^43^, ^44^, ^45^ Despite these insights, understanding ILCs in CRC remains challenging. The rarity and heterogeneity of tumour‐associated ILCs, coupled with lineage plasticity and microenvironmental reprogramming, have complicated their measurement and interpretation in human diseases.

The emergence of single‐cell RNA sequencing (scRNA‐seq)^46^, ^47^ and spatial transcriptome sequencing have enabled us to characterise the tumour microenvironment (TME) with unparalleled resolution. They exhibit powerful advantages in uncovering cellular and molecular heterogeneity and spatial architecture. Moreover, by integrating lineage tracing and trajectory inference methods, they are promised to advance our understanding of ILCs’ ontogeny, heterogeneity, and context‐dependent transdifferentiation.

Herein, we systematically investigated the heterogeneity, cellular origins, and dynamic reprogramming of ILC subsets in CRC using scRNA‐seq combined with spatial transcriptome sequencing. In addition, we delineated distinct developmental trajectories of tumour‐associated ILC populations, highlighting their different responses to the TME. Collectively, these findings not only refine our comprehension of ILC biology but also lay the groundwork for developing precision immunotherapies designed to manipulate ILC functions within the TME.

## 2 METHODS

### 2.1 Human clinical specimens

Participants were enrolled between February 2020 and August 2025 at PLA General Hospital, including 32 CRC patients and 4 healthy controls. Participants were excluded if any of the following applied: autoimmune disorders; active infectious diseases; severe hepatic or renal dysfunction; a history of any other pathologically confirmed malignancy within the preceding five years; or non‐tumour‐related emergencies, such as intestinal perforation or complete bowel obstruction, requiring urgent surgery. Clinical stages adhered to classification of the Union for International Cancer Control (UICC). Colorectal tumour tissues and matched adjacent normal intestinal mucosa were prospectively collected specifically for this study during surgical resections under the approved protocol. ‘Adjacent normal’ was defined as mucosa located ≥ 5 cm from the tumour margin and confirmed to lack malignant cells by two independent pathologists. Peripheral blood samples from CRC patients were collected specifically for research by qualified clinicians. Bone marrow aspirates were obtained from healthy hematopoietic stem cell transplatation donors. The study was approved by the PLA General Hospital Ethics Committee (S2019‐177‐01 and S2024‐523‐01) and conducted in accordance with the Declaration of Helsinki. All participants provided written informed consent, and data were anonymised.

### 2.2 Isolation of human cells from colorectal cancer tissues and blood

Fresh tumour and paired normal mucosa were cleaned, minced into 2 mm^3^ fragments, and digested in RPMI‐1640 containing 1.5 mg/mL Collagenase I and IV, 0.1 mg/mL DNase I, and 10% FBS at 37°C for 1 h. The resulting suspension was filtered through a 70‐µm mesh, centrifuged, and resuspended for downstream analysis. Peripheral blood and bone marrow samples were isolated by Ficoll density gradient centrifugation. After erythrocyte lysis, the cell pellet was resuspended for downstream applications.

### 2.3 Naïve B cell and ILC2 isolation and co‐culture

Naïve B cells were enriched from PBMCs using the EasySep™ Human Naïve B Cell Isolation Kit, following standard protocols. For the isolation of ILC2s, PBMCs were first subjected to enrichment with the Human Lineage Cell Depletion Kit, followed by FACS. The co‐culture experiment was performed in 96‐well ultra‐low‐attachment plates, where ILC2s and naïve B cells were seeded at a ratio of 1:4. The culture medium was RPMI‐1640 supplemented with 10% human AB serum, 1% penicillin‐streptomycin, 1% L‐glutamine, 1% sodium pyruvate, 1% HEPES, 1% MEM non‐essential amino acids, 0.1% 2‐mercaptoethanol (2‐ME), as well as recombinant human cytokines including IL‐2 (50 ng/mL), IL‐4 (50 ng/mL) and IL‐7 (25 ng/mL). The culture medium was replaced on day 3 of co‐culture, and cells were collected on day 6 for analysis.

### 2.4 Flow cytometry analysis or sorting

Cells were incubated with specific antibodies (30 min, 4°C). After washing, samples were briefly stained with 7‐AAD (5 min) to discriminate viability. To simultaneously detect the expression of intracellular cytokines or transcription factors, cells were enrichment of immune cells using human CD45 MicroBeads. The enriched cells were stimulated with Tonbo Cell Stimulation Cocktail for 6 h. After washing, cells were stained with a live/dead viability dye and blocked the Fc receptors, followed by incubation with antibodies for surface marker staining. The cells were then fixed and permeabilised for intracellular cytokine or transcription factor staining.

ILC2 were identified as CD45^+^Lin^−^ (CD34^−^CD19^−^CD11c^−^CD14^−^Fcer^−^) CD3^−^CD127^+^CD161^+^CRTH2^+^CD117^−^, and sorted along with ILC3 (CD45^+^Lin^−^ CD3^−^CD127^+^CD161^+^CRTH2^−^CD117), based on gating strategies shown in Figures S2B, S5, and S6C. The purity of the sorted populations was consistently > 95%.

### 2.5 Single‐cell RNA sequencing of bone marrow ILCs

Purified ILCs were held on ice in PBS/0.1% BSA, counted, diluted to the recommended of 10× Genomics Chromium v3 loading concentration for single‐cell library preparation. Sequencing was performed using the illumina platform (NovaSeq xplus).

### 2.6 Raw 10× read alignment, quality control and normalisation

FASTQ files generated from bone marrow samples were processed with Cell Ranger v5.0.1 (10× Genomics). Cellranger count mapped reads to the GRCh38 reference, performed UMI‐level quantification, and generated the gene–cell count matrix, with low‐quality reads and invalid barcodes filtered during pipeline execution. Barcode and UMI assignment were performed within Cell Ranger. To standardise sequencing depth across datasets, the aggr function within Cell Ranger was utilised with default settings. The resulting unified feature‐barcode matrix served as the input for subsequent analysis. Within the Seurat environment, raw UMI count matrices underwent rigorous quality control filtering to exclude low‐quality cells.

### 2.7 Quality control and processing of scRNA‐seq data

Raw scRNA‐seq data were handled with the Seurat R package^48^ (v.4.3.2). After importing counts into a Seurat object, quality‐control filters were applied: cells were required to exhibit ≥300 detected genes, ≥800 UMIs, and a mitochondrial UMI fraction ≤20%. Cells with abnormally high total reads (> 100000) were discarded to minimise inclusion of non–single‐cell events. Putative doublets were identified with DoubletFinder^49^ (v.2.0.3) and removed. Data integration and batch effect removal were executed via the ‘RunFastMNN’ function in Seurat‐wrappers package (v0.3.1). Prior to integration, we normalised raw expression counts (scale factor: 10000) with a standard log‐transformation and selected the top 2000 variable features.

### 2.8 Cluster‐specific differential expression analysis

Differential expression analysis relied on the Wilcoxon method implemented within Seurat's ‘FindAllMarkers’ function. Genes showing the significant differential expression (adjusted *p <* .05) were considered cluster‐specific markers.

### 2.9 CopyKat analysis to identify epithelial cell states

CopyKAt^50^ analysis delineated distinct epithelial cell states. Gene–cell matrices with gene symbols were used as input (id.type = ‘S’), and parameters were set to default values. Diploid cells automatically identified by CopyKAT were used as the reference baseline. Cells predicted as ‘aneuploid’ were defined as tumour epithelial cells, while ‘diploid’ cells were considered non‐malignant.

### 2.10 Gene set variation analysis (GSVA)

To evaluate signalling pathway activity, gene set variation analysis (GSVA) was conducted on scRNA‐seq expression matrices. Curated human gene sets were retrieved from MSigDB. The count matrix was extracted with the GetAssayData function. GSVA (v.1.52.2) was applied to the count matrix with the parameters ‘kcdf = Gaussian’ and ‘method = zscore’. After computing pathway z‐scores per cell, we added the resulting data to the Seurat object to enable further analysis. Significant pathways for each cluster were determined via the ‘FindAllMarkers’ function (Wilcoxon test), utilising significance cutoffs of an adjusted *p*‐value < .05 and a log_2_ fold change ≥ 0.3.

### 2.11 Pearson's correlation analysis

**FIGURE 1:**
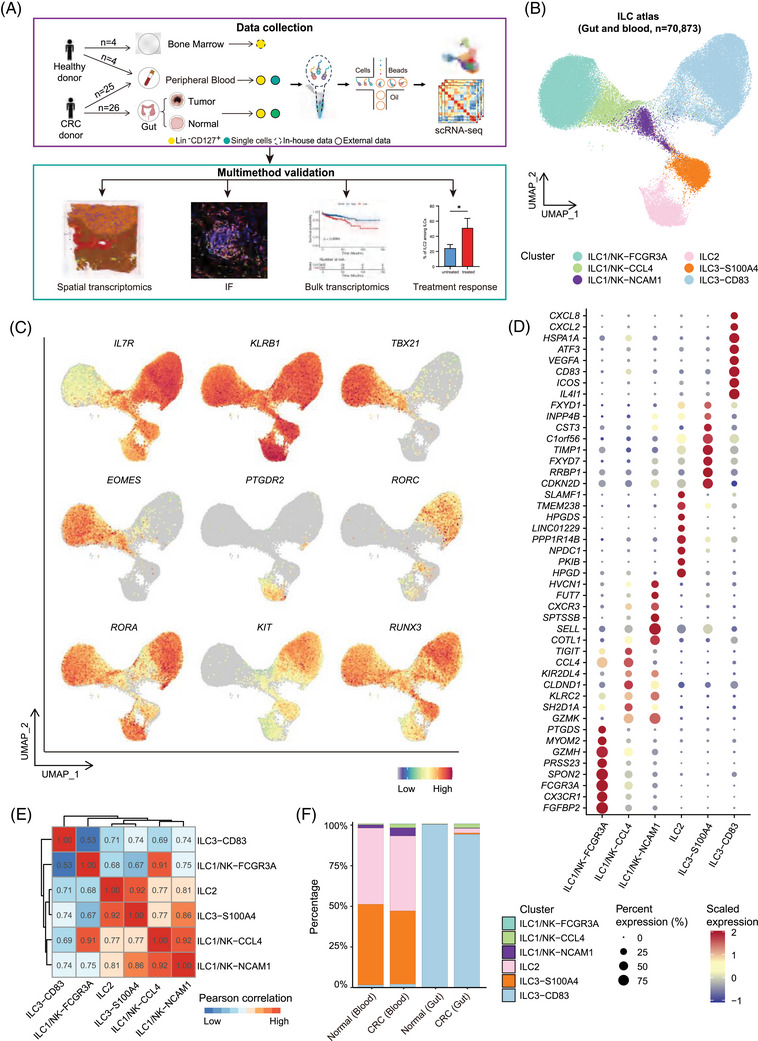
Heterogeneity of ILC populations in CRC tumour and peripheral blood. (A) Schematic overview of analytical workflow and experimental design. (B) Uniform manifold approximation and projection (UMAP) visualisations of ILCs. Colours encode cell types. (C) UMAP plots showing the expression of representative genes associated with the identified clusters. Colours represent the expression levels of genes. (D) Bar plots showing the proportions of six ILC clusters across groups from the HRA000919 dataset. Colours encode cell types. (E) Heatmap showing the Pearson's correlation across ILC clusters. Colours encode the coefficient of Pearson's correlation. (F) Dot plots showing the top 8 differentially expressed genes (DEGs) of each cluster. Colours represent the expression levels of genes and dot size encodes the proportion of gene‐expressing cells.

**FIGURE 2:**
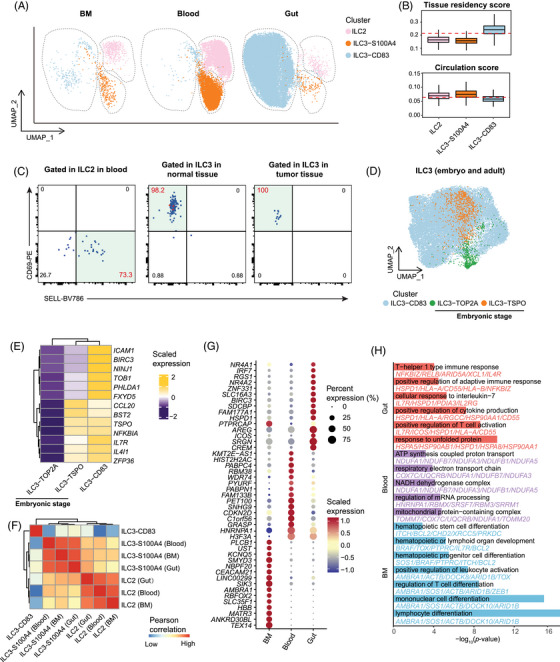
Tissue distribution and cellular origins of ILCs in CRC patients. (A) UMAP plots showing the distribution of ILC clusters across BM (healthy donors only), blood (healthy donors and CRC patients), and gut (healthy donors and CRC patients). (B) Boxplots showing the score of the tissue residency and circulation. Centre line indicates the median value, lower and upper hinges represent the 25th and 75th percentiles, respectively, and whiskers denote 1.5 interquartile range. Colours encode cell types. (C) Flow cytometry of the expression of *SELL* and *CD69* in ILC2s from peripheral blood (left), and ILC3s from normal tissue (middle) and tumour tissue (right). (D) UMAP plots showing the distribution of the fetal and adult ILC3 clusters in the intestine. (E) Heatmap of the expression levels of selected genes in each cell type. Colours represent expression levels of genes. Representative genes are listed. (F) Heatmap illustrating the Pearson's correlation across ILC2, ILC3‐S100A4 from different sites, and ILC3‐CD83. Colours encode the coefficient of Pearson's correlation. (G) Dotplots showing the top 15 DEGs of ILC3‐S100A4 across different tissue sites. Colours represent the expression levels of genes and dot size encodes the proportion of gene‐expressing cells. (H) Bar chart showing the Gene Ontology Biological Process (GOBP) enrichment analysis of upregulated DEGs in ILC3‐S100A4 from BM, blood, and gut. Bars represent –log_10_(*p*‐value) of enriched terms.

To evaluate transcriptional similarity within and across ILC populations, we performed Pearson's correlation analysis using the top 2000 HVGs. In integration analysis section, we conducted Pearson's correlation analysis on the integrated dataset using mutual nearest neighbour (MNN) embeddings in Figures 1E and 2E and S2E.

### 2.12 Enrichment analysis

To investigate functional differences among ILC clusters, we conducted Gene Ontology (GO) enrichment via the clusterProfiler^51^ R package (v.4.6.2). Significant terms were determined from DEGs using the ‘compareCluster’ command with default arguments. Enriched GO terms returned by ‘compareCluster’ were converted to a data frame. We computed the enrichment score as –log_10_(*p*.adjust) and visualised the results as horizontal bar plots using ggplot2 (v.3.5.1).

### 2.13 MiloR

We applied the miloR R package^52^ (v.2.0.0) to evaluate differential cell abundance across overlapping neighbourhoods of approximately 50–200 cells. For each condition (tumour and normal), analysis was restricted to the 3000 genes exhibiting the highest variability among single cells. PCA was performed on this submatrix, and the result was passed into a miloR object for further analysis. The ‘buildGraph’ function in miloR was performed with parameters *k* = 30 and *d* = 50, followed by ‘makeNhoods’ and ‘calcNhoodDistance’ pipeline. Using the ‘testNhoods’ function in miloR, we assessed differences in cell abundance between treated and control samples across neighbourhoods. The resulting *p*‐values were adjusted by miloR to generate spatial FDR estimates that incorporate neighbourhood connectivity, as illustrated in Figure 5A and B.

### 2.14 Cell type enrichment analysis

Tissue enrichment of ILC meta‐clusters was assessed by calculating odds ratios (OR)^53^, ^54^ from 2×2 contingency tables for each cluster–tissue pair. Significance was determined via Fisher's exact test, with *p*‐values corrected using the Benjamini–Hochberg method.

### 2.15 Developmental trajectory prediction

CytoTRACE was first applied to the QC‐filtered raw UMI count matrix. To reduce sparsity, genes detected in ≥5 cells were retained. CytoTRACE was executed with multithreading (ncores = 25) to compute a per‐cell differentiation score (Figure 4B), where higher values indicate less differentiated states. Scores were min–max scaled to [0, 1]. Cells in the top decile of the CytoTRACE score were designated as putative progenitor/root candidates and used to initialise lineage roots and directionality in downstream trajectory inference. Developmental Trajectories were then inferred with Slingshot.^55^

### 2.16 Calculation of signature score and gene set definitions

Activity scores were generated via AUCell (v1.22.0). This algorithm derives AUC‐based enrichment values from gene rankings within individual cells, operating independently of expression normalisation. Rankings were generated with ‘AUCell_buildRankings’, and AUC values were obtained using ‘AUCell_calcAUC’. To evaluate functional variations among ILC subsets, we applied gene sets related to residency, circulation, and stress response state^56^ as defined in previous studies.

### 2.17 SCENIC analysis

Regulatory inference was executed via the SCENIC pipeline.^57^ This analysis identifies co‐expressed gene modules and infers the activity of transcription factors regulating these modules. First, gene expression data were processed and normalised using the Seurat pipeline. PySCENIC initiated network reconstruction by applying GRNBoost2 to identify co‐expression modules. The AUCell algorithm generated target enrichment scores for each cell, which were stored in the Seurat structure. Significant regulons (adjusted *p*‐value < .05) were plotted via pheatmap and ggplot2.

### 2.18 Cell2location

Cell2location^58^ was applied to integrate single‐cell–derived reference signatures with spatial transcriptomics and to estimate location‐specific abundances of each cell type. Spatial deconvolution was performed by aligning single‐cell data with spatial references. The analysis was guided by cell‐type‐specific markers, which allowed for accurate assignment of cells to their respective tissue locations. The spatial distributions of cell types were visualised using spatial heatmaps.

### 2.19 MistyR

We used mistyR^59^ (v1.10.0) to explore the relationships between spatial organisation and cell‐state heterogeneity in immune and stromal compartments. As input, we used the cell2location across all Visium slides. Three spatial contexts (views) were modelled: (1) Intrinsic view, representing the relationships among deconvolution estimates within each spot; (2) Juxta view, aggregating estimates from immediate neighbouring spots (distance ≤ 3); (3) Para view, weighting contributions from more distant neighbours within a radius of 5 spots. For each slide, mistyR estimated the contribution (‘importance’) of each predictor view. Importances were standardised and aggregated (median across slides), and interpreted as evidence of the predicted dependencies between different cell types to investigate the potential interactions between them (Figure 6D).

### 2.20 Survival analysis

Survival assessment was conducted using the R packages survival (v3.5.7) and survminer (v0.4.9). Cases from TCGA and GEO were dichotomised at the optimal cut point of the signature module score identified by the ‘surv_cutpoint’ function. Kaplan–Meier survival curves were generated, with significance evaluated via log‐rank testing (two‐sided *p*  < .05) (Figure 7A).

### 2.21 ROC analysis

We assessed the model's discriminative performance using ROC methodology. Predicted class probabilities were passed to pROC. ROC curves were obtained by estimating sensitivity and 1 – specificity across threshold grids using the ‘roc()’ function. Discrimination was summarised by the area under the curve (AUC) computed. Curves were visualised with ‘plot.roc’.

### 2.22 Immunofluorescence

Human samples were collected from PLA General Hospital and fixed in 4% paraformaldehyde and then used for paraffin embedding. Chilled embedded tissue blocks were sectioned at thickness of 5 µm. Routine deparaffinisation, rehydration and blocking of the slides were performed. Primary antibodies were then paved onto section slides overnight at 4°C, and then secondary antibodies were used to incubated the slides for 30 min at room temperature. Finally, signal amplification was performed with DendronFluor TSA (NEON 4‐color IHC Kit, Histova; NEFP450; 1:100; 20–60 s). Between each step, 3 times PBS rinses were performed to clean the slides. For multi‐antibodies staining, each antigen was then labelled in sequence and antigen retrieval and blocking were performed before incubation of next primary antibody. Nuclei were counterstained with DAPI, and images were acquired on a confocal microscope (Nikon Ti‐E A1 or ZEISS LSM 880). Primary antibodies used are listed in Table S1.

### 2.23 Statistics

Statistical analyses were conducted in R (version 4.3.2). Depending on distributional assumptions, we applied Student's *t*‐test or the Wilcoxon rank‐sum test. Results were considered significant at *p* <.05 (ns, *p* > .05; *p < .05; **p < .01; ***p < .001; ****p < .0001).

## 3 RESULTS

### 3.1 Heterogeneity of ILC populations in CRC tumour and peripheral blood

To define cell diversity of ILC populations in CRC, we profiled a comprehensive CRC scRNA‐seq atlas by including several single‐cell studies.^60^, ^61^, ^62^ These data cover the unbiased cell map, which includes a rich population of ILC1/NK cells, as well as studies on the abundant helper ILC cell populations (Lin^−^ CD127^+^; Lin: TCRγδ^−^ TCRαβ^−^ CD3^−^ CD19^−^ CD14^−^ CD16^−^ CD94^−^ CD123^−^ CD34^−^ CD303^−^ FcεRI^−^) from CRC, adjacent tissues, and peripheral blood. Additionally, scRNA‐seq data from peripheral blood from healthy donors were included as controls. A graphical representation of the study design is provided in Figure 1A. After rigorous quality control, a total of 93 546 cells from 42 samples were included for further analysis. Based on the characteristic genes reported in prior studies, we classified the 93 546 single cells into 14 major cell types (Figure S1A), including B cells (*MS4A1*, *CD79A* and *JCHAIN*), T cells (*CD3D*, *CD3E*, *CD4*, *CD8A* and *CD8B*), ILCs (*KLRB1* and *IL7R*), monocytes (*CD14*, *CD1C* and *FCN1*), macrophages (*CD68*, *C1QA* and *C1QC*), dendritic cells (*CLEC10A*, *CD1C* and *FCER1A*), mast cells (*CPA3* and *TPSAB1*), granulocytes (*FCGR3B* and *CSF3R*), endothelial cells (*PLVAP* and *VWF*), fibroblasts (*COL1A1*), Schwann cells, pericytes (*RGS5*), smooth muscle cells (*ACTA2*) and epithelial cells (*EPCAM*) (Figure S1B). We applied CopyKAT to identify neoplastic epithelial cells by inferring chromosomal aneuploidy from scRNA‐seq data (Figure S1C).

Through focusing on ILC populations, we further identified six distinct ILC subclusters namely ILC1/NK‐FCGR3A, ILC1/NK‐CCL4, ILC1/NK‐NCAM1, ILC2, ILC3‐S100A4 and ILC3‐CD83, based on the classical ILC taxonomy and transcriptomic signatures of feature genes (Figures 1B and S1D). All six ILC clusters express feature genes of ILCs, such as *IL7R* and *KLRB1*. The three ILC1/NK clusters display high expression of ILC1/NK signature genes *TBX21* and *EOMES*. ILC2s were distinguished by specific expression of *PTGDR2* (encoding CRTH2). ILC3s exhibited higher expression of *RUNX3*, *KIT*, *RORA*, and *RORC* (Figure 1C). These helper ILC populations were primarily derived from an ILC‐sorted dataset (Figure S1E).

Notably, each ILC subset exhibited distinct gene expression signatures. In particular, the ILC1/NK‐FCGR3A subset exhibits the highest cytotoxic score (Figure S1F), characterised by the distinctive expression of cytotoxic molecules, including *FCGR3A* and *GZMH*. In contrast, the ILC1/NK‐CCL4 and ILC1/NK‐NCAM1 subclusters, while retaining cytotoxic features, are predominantly characterised by the expression of immune‐regulatory genes. Specifically, the ILC1/NK‐NCAM1 subcluster expresses *CXCR3* and *SELL*, while the ILC1/NK‐CCL4 subcluster is characterised by immune checkpoint gene *TIGIT* and *CCL4*. These molecular signatures might underscore their primary involvement in immune‐regulatory processes, rather than direct cytotoxic activity. Interestingly, we find that ILC2 and ILC3‐S100A4 share similar transcriptional profiles. Specifically, they exhibit overlapping gene expression patterns linked to key cellular processes, including cell cycle regulation, protein processing, and ion transport. However, ILC2s distinguish themselves by the distinctive expression of a set of genes, such as *HPGD*, *HPGDS*, *PKIB*, *NPDC1*, and *SLAMF1*. These specifically expressed markers orchestrate essential biological activities, ranging from prostaglandin metabolism, intracellular signalling, to immune regulation. On the other hand, ILC3‐CD83 stands out with its robust expression of a diverse array of immune‐modulatory genes, including *ICOS*, *CD83*, *CXCL2*, *CXCL8*, *VEGFA*, and *ATF3*. These genes are associated with pathways involved in immune activation, driving inflammatory responses, and facilitating angiogenesis (Figure 1D and E).

By analysing ILC1/NK enriched dataset, we found that three clusters of ILC1/NK cells showed increased proportions in tumours compared with normal tissues and accounted for a relatively large proportion among ILCs (Figure S1G). Therefore, we focused on the ILC‐sorted dataset and further assessed the variation in the proportions of helper ILCs (ILC2s and ILC3s in our data) across different patient tissues. Notably, we observed higher proportions of ILC2 and ILC3‐S100A4 cells in peripheral blood compared to intestinal tissue, whereas ILC3‐CD83 cells were more abundant in the latter. Although there was no difference in the proportions of ILC2 and ILC3‐S100A4 in the peripheral blood of healthy donors and CRC patients, compared to normal intestinal tissues, ILC2 and ILC3‐S100A4 were mainly enriched in CRC. Additionally, as the principal resident subset in intestinal tissue, ILC3‐CD83 was significantly reduced in CRC tumours, consistent with previous findings^24^, ^63^, ^64^ (Figures 1F and S1H).

### 3.2 Tissue distribution and cellular origins of ILCs in CRC

While some understanding of the tissue origin of ILC1/NK in tumours has been established,^65^ knowledge regarding the origins of ILC2s and ILC3s remains relatively limited. To elucidate the origins and distribution patterns of ILC2s and ILC3s, we integrated and conducted a comprehensive analysis of ILC populations derived from BM, peripheral blood, and gut tissues of healthy donors (Figure 2A). We observed that ILC3‐CD83 is predominantly distributed in intestinal tissues, while its proportion in bone marrow and peripheral blood is relatively low (Figures 2A and S2A), suggesting that it may serve as the gut tissue‐resident population. This distribution pattern is consistent with tissue‐residency signature scores, wherein ILC3‐CD83 demonstrates higher tissue‐residency scores and lower circulatory scores (Figure 2B). Consistent with this pattern, flow‐cytometric analysis showed that the circulation‐associated marker SELL was expressed at substantially higher levels in ILC2 from peripheral blood compared with ILC3 in normal tissue and tumour tissue, whereas the tissue‐residency marker CD69 displayed the opposite trend (Figure 2C). We hypothesise that these ILC3‐CD83 cells might originate during embryonic stages and colonise the intestinal tract, where they subsequently persist in adulthood. To further confirm this hypothesis, we performed integrative analysis between ILC3‐CD83 in CRC and ILC3s in embryonic intestine tissue spanning from week 8 to week 12. In embryonic intestinal tissue, two transcriptionally distinct ILC3 clusters were identified namely ILC3‐TOP2A and ILC3‐TSPO (Figure S2C). ILC3‐TOP2A was characterised by a proliferative profile with high expression of cell proliferative genes such as *TOP2A* and *CDK1*. ILC3‐TSPO exhibited high expression level of immune‐regulatory genes such as *IL7R* and *IL4I1* and broad enrichment of immune‐related functional pathways (Figure S2D and E). Integrative analysis showed that ILC3‐CD83 merge well with ILC3‐TSPO cluster on the UMAP plot (Figure 2D) with the shared expression of the immune‐regulatory genes, such as *ZFP36*, *BST2*, *CCL20* and *NFKBIA* (Figure 2E). This is also in agreement with the correlation analysis result, in which ILC3‐CD83 display higher similarity with ILC3‐TSPO than with ILC3‐TOP2A (Figure S2F).

ILC2 and ILC3‐S100A4 were predominantly present in both bone marrow and peripheral blood, with a lower proportion found in the gut (Figures 2A and S2A). This was in line with the higher score of circulation signature in ILC2 and ILC3‐S100A4 (Figure 2B). As expected, ILC2s derived from BM, peripheral blood, and the intestinal tract exhibited high transcriptional similarity, and the same pattern was observed in ILC3‐S100A4 cells (Figure 2F). These observations suggest an association between ILC2 and ILC3‐S100A4 in intestinal tissues and the BM, with the possibility that these cells may migrate from the BM to the intestinal tract via the peripheral blood. We further compared these bone marrow‐derived ILCs (ILC2 and ILC3‐S100A4) across tissue compartments. We found that bone marrow ILC3‐S100A4 cells displayed higher expression of transcriptional features typical of immature or proliferative states, with prominent expression of genes such as *TEX14* and *MATR3*. GO analysis revealed an enrichment of pathways related to lymphocyte differentiation and immune cell development in bone marrow ILC3‐S100A4. Peripheral blood ILC3‐S100A4 cells exhibited a distinct transcriptional shift towards metabolic activation, with enrichment of pathways related to ‘regulation of mRNA processing’, ‘respiratory electron transport chain’, and ‘ATP synthesis coupled proton transport’. Compared with ILC3‐S100A4 cells in BM and peripheral blood, those in the gut displayed elevated the expression of immunoregulatory genes, such as *ICOS*, *SRGN*, *NR4A1*, and *NR4A2*, and showed enrichment of pathways associated with T cell activation and Th‐1 type immune responses (Figure 2G and H). Similarly, ILC2s showed distinct transcriptional profiles between BM and gut (Figure S2G and H). These consistent and location‐specific signatures across ILC subsets may reflect tissue‐specific adaptations, though whether these differences arise from microenvironmental imprinting remains to be determined.

### 3.3 Heterogeneity of tissue‐resident ILC3 populations in CRC

**FIGURE 3:**
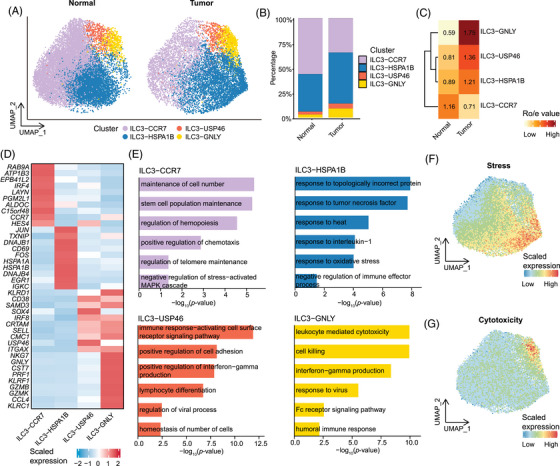
Heterogeneity of tissue‐resident ILC3 populations in CRC. (A) UMAP plots showing the distribution of ILC3 (ILC3‐CD83 cluster in Figure 2A) under different conditions. (B) Bar plots showing the proportions of four ILCs clusters across group. Colours encode cell types. (C) Tissue preference of each cluster estimated by the STARTRAC‐dist index. Colours represent the STARTRAC‐dist value (Ro/e). (D) Heatmap showing the top 10 DEGs of each cluster. Colours represent the expression levels of genes. (E) Bar chart showing the GOBP enrichment analysis of upregulated DEGs in ILC3‐CCR7, ILC3‐HSPA1B, ILC3‐USP46, and ILC3‐GNLY. Bars represent –log_10_(*p*‐value) of enriched terms. (F) UMAP plots showing the module score enrichment of cytotoxicity gene signatures across different cell populations. (G) UMAP plots showing the module score enrichment of stress gene signatures across different cell populations.

Tissue‐resident ILC3s play important roles in maintaining mucosal immunity, yet their heterogeneity remains largely unexplored. To fill this knowledge gap, we further focus on the tissue‐resident ILC3‐CD83. Unsupervised clustering further identified four transcriptionally distinct subgroups, namely ILC3‐CCR7, ILC3‐HSPA1B, ILC3‐USP46 and ILC3‐GNLY (Figures 3A and S3A). These subgroups showed marked differences in transcriptome profile and cell distribution. The ILC3‐CCR7 was predominantly localised in the normal intestinal mucosa, with high expression of genes such as *RAB9A*, *IRF4*, and *CCR7*, all of which have been reported to be involved in immune cell signalling and cell migration. Pathway enrichment analysis revealed that the ILC3‐CCR7 cluster enriched GO terms related to ‘maintenance of cell number’, ‘stem cell population maintenance’, and ‘regulation of haematopoiesis’. These observations indicate a potential involvement of ILC3‐CCR7 in the preservation of intestinal stability and the subtle regulation of immune responses. Intriguingly, unlike ILC3‐CCR7, which is enriched in the normal intestine, the other three subsets of ILC3s (ILC3‐USP46, ILC3‐GNLY and ILC3‐HSPA1B) are primarily distributed in CRC. These results indicated that tissue‐resident ILC3 underwent obvious remodelling in the CRC context (Figure 3B). As the most prominent tissue‐resident ILC3 subsets in tumour, ILC3‐HSPA1B is distinguished by upregulated expression of cell stress‐related genes (e.g., *HSPA1B*, *HSPA1A*, and *JUN*), all of which have been reported to be involved in cellular stress responses and adaptive mechanisms.^56^, ^66^ Enrichment analysis revealed that ILC3‐HSPA1B cluster was enriched for stress‐related GO terms, such as ‘response to heat’, ‘response to interleukin‐1′, ‘response to oxidative stress’, and ‘response to tumour necrosis factor’. Additionally, this population exhibited the highest scores of stress response (Figure 3F), further highlighting the unique role of the ILC3‐HSPA1B group in cellular stress response. Notably, this cluster was enriched for GO terms associated with the ‘negative regulation of immune effector processes’ and low activity across immune‐related pathways, suggesting its potential immunosuppressive role (Figure S3C). ILC3‐USP46 displayed elevated expression of genes involved in cell adhesion and migration (e.g., *SELL* and *CD38*), and transcriptional regulation of immune responses (e.g., *IRF8*). Additionally, ILC3‐USP46 enriched immune‐regulation‐related GO terms, such as ‘immune response‐activating cell surface receptor signalling’, ‘positive regulation of interferon‐gamma production’, ‘lymphocyte differentiation’, indicating its proinflammatory function. The ILC3‐GNLY subset exhibited a transcriptional program strongly enriched for immune effector pathways, including ‘leukocyte‐mediated cytotoxicity’, ‘cell killing’, and ‘interferon‐gamma production’. Consistent with these features, ILC3‐GNLY showed markedly elevated scores in a cytotoxicity‐associated gene signature (Figure 3G), indicative of the cytotoxic phenotype.

### 3.4 Cell fate transition of tissue‐resident ILC3s under CRC

**FIGURE 4:**
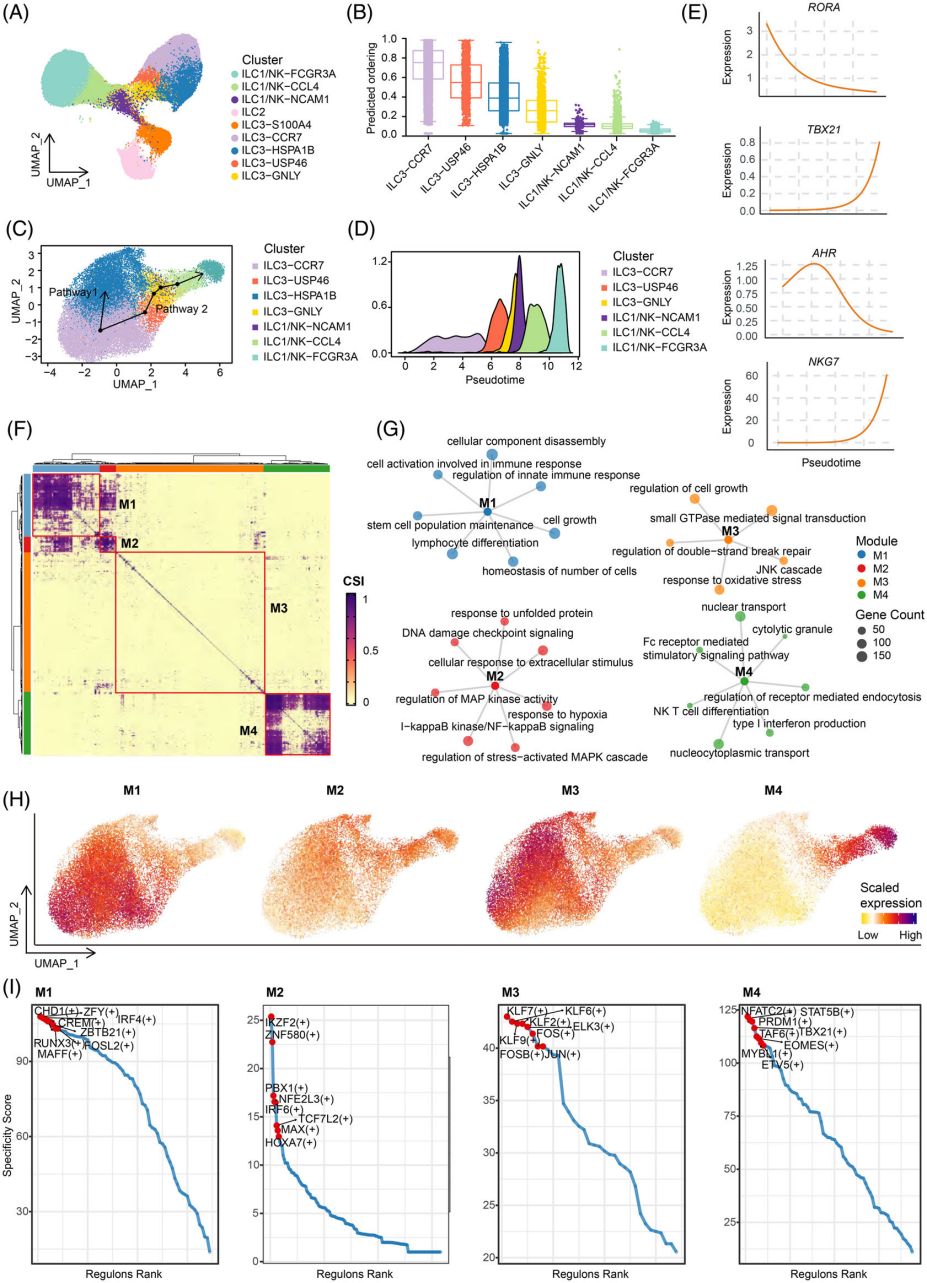
Cell fate transition of tissue‐resident ILC3 populations in CRC. (A) UMAP plots showing ILCs cluster in the intestine. Colours encode cell types. (B) Boxplots showing CytoTRACE‐predicted differentiation potential of sorted ILC subtypes (higher scores indicate less differentiation). Centre line indicates the median value, lower and upper hinges represent the 25th and 75th percentiles, respectively, and whiskers denote 1.5 interquartile range. Each data point represents one sample. (C) Pseudotime trajectory analysis reveals the lineage relationships among different ILC subsets using Slingshot. (D) Ridge plots showing the density distribution of ILC subsets along pseudotime trajectories in pathway 1. (E) Pseudotime plot displaying the kinetics of indicated genes (RORA, TBX21, AHR, NKG7) from the root of the trajectory to pathway 2. Each point corresponds to a single cell. (F) Heatmap showing four transcription factor (TF) modules (M1‐4) identified by hierarchical clustering based on the regulon connection specificity index (CSI). Regulons were calculated using SCENIC analysis. Colours encode CSI between regulons. (G) The correlation network of TF regulons for the four regulon modules. (H) UMAP plots showing the four regulon modules. (I) The top eight regulons in modules in F are highlighted in red and labelled on the plot. The specificity score is shown on the y axis.

Analogous to T helper cells, ILCs exhibit context‐dependent plasticity driven by signals from the tissue microenvironment, including cytokines, lineage defining transcription factors, and changes in chromatin accessibility.^16^, ^67^, ^68^ However, whether this property shapes ILC3 states in colorectal cancer remains unclear. In our data, ILC3‐GNLY subset has been observed to exhibit transcriptional features traditionally associated with cytotoxic activity. When mapping all the four tissue‐resident ILC3 subpopulations to ILCs immune landscape, we noted a significant spatial proximity between the ILC3‐GNLY subset and cytotoxic ILC1/NK‐associated clusters, such as ILC1/NK‐CCL4 and ILC1/NK‐NCAM1 (Figure 4A). This spatial relationship prompted us to investigate whether ILC3‐GNLY may represent a distinct ILC3 subset poised for conversion to a cytotoxic ILC1/NK‐like phenotype. Using CytoTRACE method, we inferred the relative differentiation states of ILC3 and ILC1/NK clusters. Notably, ILC3‐associated clusters exhibited greater stemness/progenitor potential compared to ILC1/NK‐like clusters. Among them, the ILC3‐CCR7 showed the greatest differentiation potential, indicating its immature identity (Figure 4B). Based on these findings, ILC3‐CCR7 was selected as the root cluster for subsequent pseudotime trajectory analysis. Trajectory inference analysis using slingshot revealed two putative differentiation pathways starting from ILC3‐CCR7 in the gut (Figure 4C). In one branch, differentiation proceeds toward the ILC3‐HSPA1B subset. In the other branch, ILC3‐CCR7 cells progress through intermediate states, including ILC3‐USP46 and ILC3‐GNLY, towards transcriptional profiles resembling ILC1/NK cells (Figure 4C and D). This putative ILC3‐ILC1 transform trajectory is accompanied by a progressive downregulation of classical ILC3 transcription factors, such as *RORA* and *AHR*, along with a stepwise upregulation of genes associated with ILC1/NK effector functions, including *TBX21* and *NKG7* (Figure 4E). By analysing RNA‐seq data of purified ILC3 populations from published study, we found that ILC3 from adjacent  tissues expressed stronger ILC3‐related signatures, whereas tumour‐derived ILC3 displayed relatively higher ILC1‐related signatures (Figure S4A). These observations suggest intestinal ILC3 display increased expression of ILC1‐associated signatures under tumour microenvironmental conditions, aligning with prior reports of ILC3 plasticity in intestinal inflammation.^28^ Nevertheless, lineage tracing and functional experiments will be necessary to definitively determine whether a bona fide ILC3‐to‐ILC1 conversion occurs in the CRC microenvironment.

To dissect the regulatory networks governing ILC plasticity, we applied SCENIC analysis to identify transcription factor module. Four transcription factor modules (M1‐M4) were readily identified based on unsupervised clustering analysis of transcription factor regulons (Figure 4F–H). M1, which is most highly expressed in the ILC3‐CCR7 subset, is enriched for processes maintaining homeostasis, such as ‘homeostasis of number of cells’, ‘stem cell population maintenance’, and ‘cellular component disassembly’. In contrast, M2 and M3 together define a stress‐ and injury‐responsive transcriptional program in ILC3, marked by activation of the ‘DNA damage checkpoint signalling’, ‘I‐kappaB kinase/NF‐kappaB signalling’, ‘JNK cascade’ and ‘small GTPase‐mediated signal transduction’. Specifically, the expression level of M3 is higher on pathway 1, and it contains several transcription factors related to cellular stress, such as JUN and FOSB. This indicates that these factors play a crucial role in the process of cells transforming into the ILC3‐HSPA1B phenotype. We further validated that ILC3‐HSPA1B subset, which predominantly constitutes pathway 1, exhibited increased expression of cell stress gene FOS at the protein level as measured by flow cytometric analysis (Figure S4C). Finally, M4 activated in intermediate and late‐stage subsets along ILC3‐ILC1 transform pathway. Its enriched GO terms associated with immune effector functions, in line with high activity of TBX21 and EOMES, which specify type‐1 identity and cytotoxic potential. Importantly, NFATC2 is involved in M4 module, which has been reported to open the transcriptional program for cytotoxic effector genes such as *IFNG* and granzymes.^69^ In addition, the M4 module contains STAT5B, a transcriptional mediator downstream of IL‐2/IL‐15 signalling. This factorthat was well known for its critical roles in promoting cell proliferation, survival, and the induction of the perforin‐granzyme machinery.^70^, ^71^ In parallel, PRDM1 has been reported to consolidate terminal effector fate by locking in ILC1/NK‐like identity and restraining excessive cytokine production to prevent uncontrolled inflammation.^72^, ^73^ Flow cytometric analysis showed that ILC3‐GNLY cells could produce IFN‐γ and express EOMES (Figure 4SB), consistent with a type‐1 effector profile associated with the M4 module. Together, these observations suggest that the M4 module is associated with acquisition of type‐1 effector characteristics, as evidenced by both transcriptional signatures and protein‐level validation, though the precise regulatory mechanisms and the extent of phenotypic conversion require further investigation.

### 3.5 Location and potential function of tumour‐associated ILC2s

**FIGURE 5:**
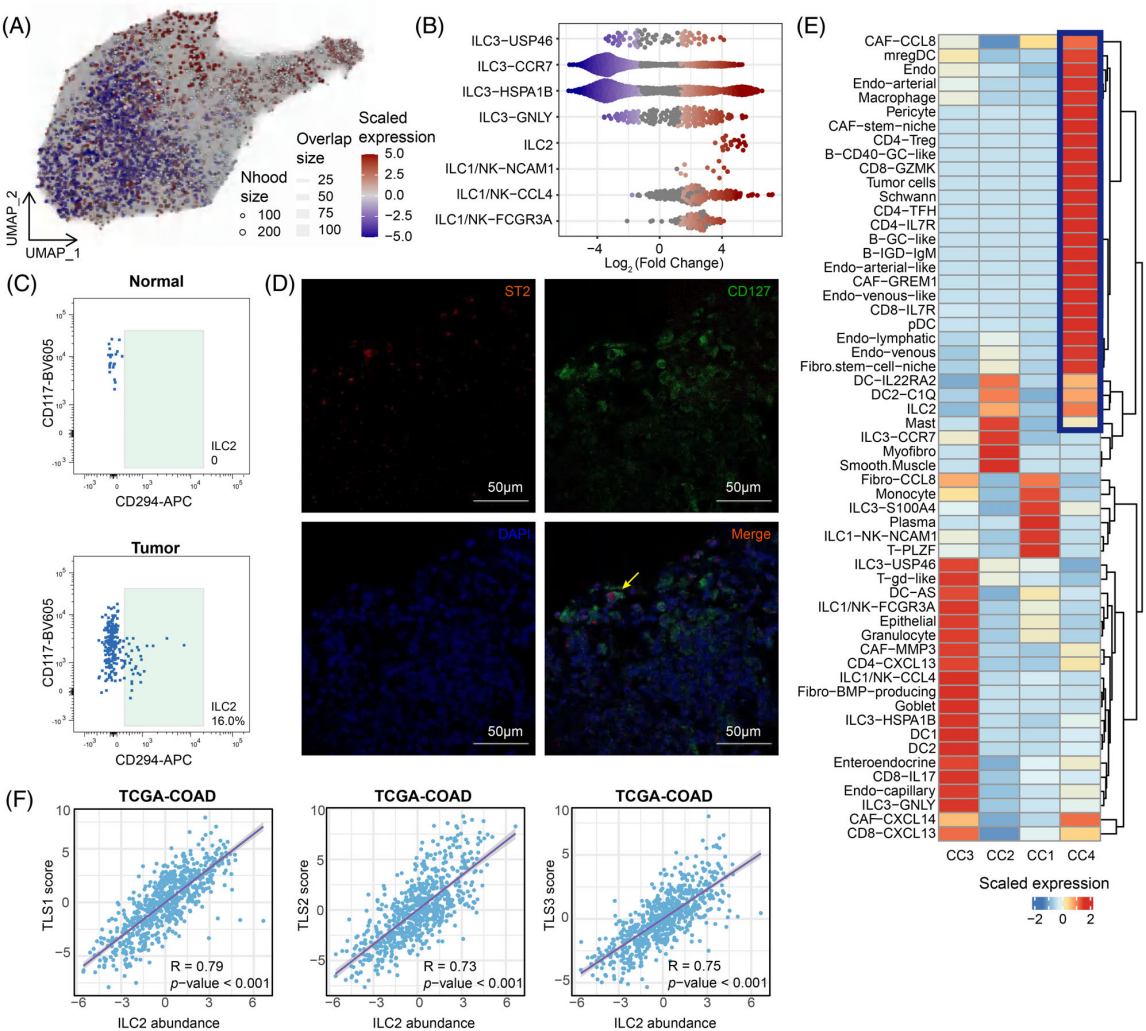
Characterisation of the tissue organisation of tumour‐associated ILCs. (A) UMAP plots showing overlapping neighbourhoods of cells, as calculated using MiloR. Red and blue colours indicate neighbourhoods with significant enrichment for tumour tissue (red) or normal tissue (blue). The point size indicates the number of cells in a neighbourhood, and the edge thickness indicates the number of cells shared between pairs of neighbourhoods. (B) Beeswarm plot displaying the distribution of log fold change in abundance between tumour tissue (red) or normal tissue (blue) in neighbourhoods from different cell types. Differential abundance neighbourhoods at FDR 10% are coloured. (C) Flow cytometry analysis of ILC2 subsets from normal intestinal mucosa and CRC tissues. (D) Representative example of ILC2s in CRC tissues stained by multiplex immunohistochemistry. Stains: ST2 (red), CD127 (green) and DAPI (blue). Scale bar: 50 µm. (E) Scaled median cell‐type compositions within each spatial niche. Colours indicate the relative abundance of each cell type within each niche. (F) Abundance estimation and relationship of tertiary lymphoid structures (TLS) signature (TLS signatures 1^77^2^78^ and 3^79^) and ILC2 signature in spatial transcriptomics. Correlation was evaluated using the two‐sided Spearman rank correlation coefficient.

Numerous investigations emphasise that ILCs exert a profound influence on oncogenic onset, malignant evolution, and eventual clinical outcomes via a multitude of straightforward and mediated pathways.^18^, ^24^, ^74^ The biological impact of these innate populations is strictly dictated by the specific temporal phase of tumourigenesis alongside the prevailing state of host adaptive immunity.^75^, ^76^ We further conducted MiloR analysis to identify tumour‐associated ILC populations in gut tissues. Interestingly, a striking contrast in the distribution of ILC2s was observed between normal and tumour tissues. In tumour tissues, the dramatic enrichment of ILC2s was observed, whereas ILC2s were nearly absent in normal tissues (Figures 5A and B). This observation was further substantiated by both flow cytometric analysis and immunofluorescence (IF) staining, which confirmed the enrichment of ILC2s in tumour tissues (Figure 5C and D).

As the innate counterparts of CD4^+^ T helper cells, ILCs primarily modulate immune responses by secreting cytokines that influence other immune cells, unlike cytotoxic cells. Consequently, a thorough characterisation of the topographical and structural attributes within the ILC‐associated niche is warranted to decipher their contribution to colorectal malignancy progress. We further collected six spatial transcriptomics datasets, including two normal and four CRC samples. We first calculated cell type constitution for each spot of every slice based scRNA‐seq reference using cell method. Then, unsupervised clustering analysis of spots based on cellular composition identified four distinct microenvironmental niches (CC1‐CC4). These niches exhibited diverse cellular composition landscapes. Notably, in CC4, we observed co‐localisation of ILC2s with cells associated with tertiary lymphoid structures (TLS), including CD4^+^ Tfh cells, B‐CD40‐GC‐like cells and tumour cells (Figure 5E).

**FIGURE 6:**
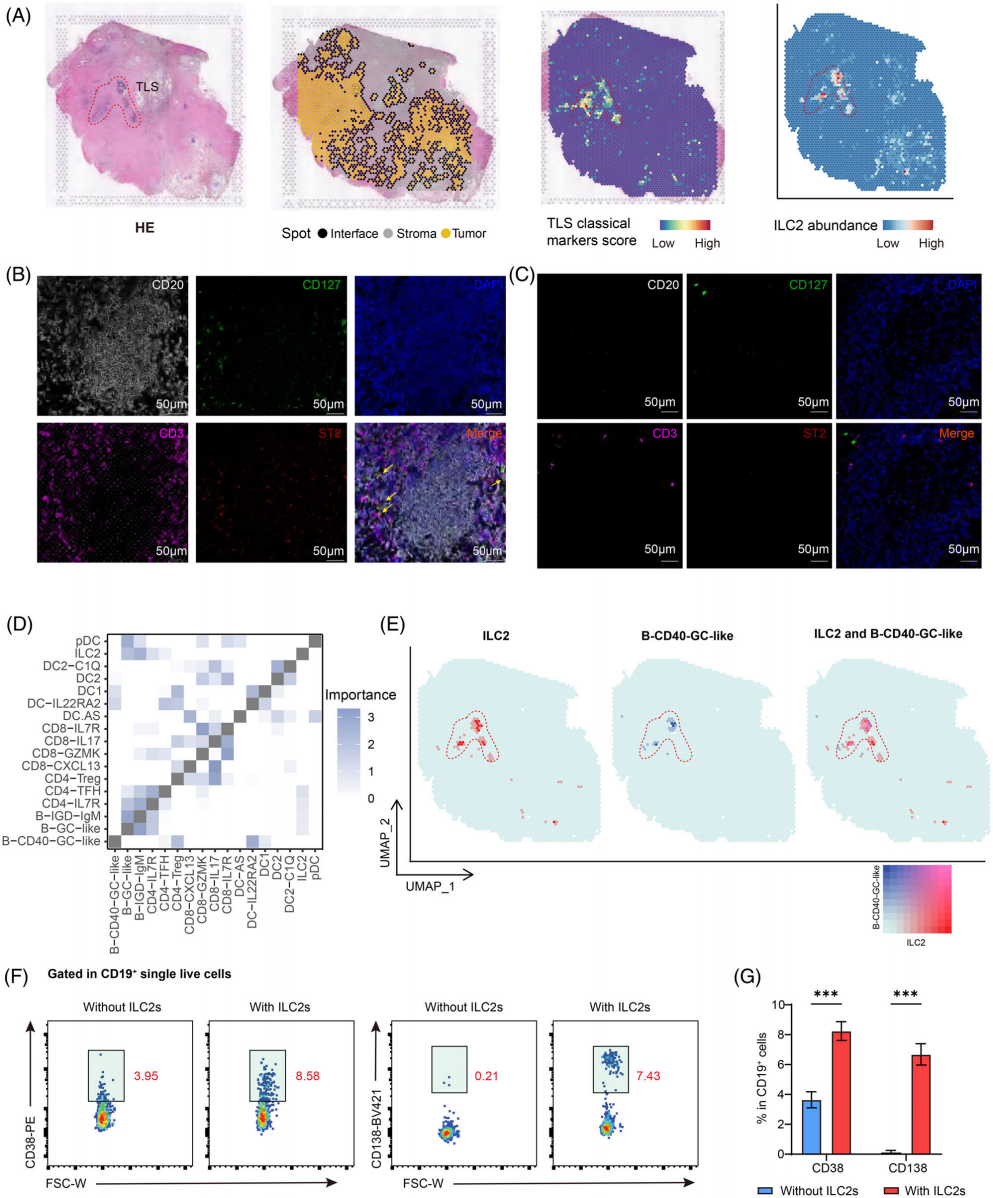
Location and potential function of tumour‐associated ILC2s. (A) H&E staining, the abundance estimation and relationship of TLS signature and ILC2 signature in spatial transcriptomics section from P2. (B) Representative multiplex immunofluorescence images showing the distribution of ILC2s in TLS regions. Stains: CD20 (white), CD3 (purple), CD127 (green), ST2 (red), and DAPI (blue). Scale bar: 50 µm. (C) Representative multiplex immunofluorescence images showing the distribution of ILC2 in non‐TLS regions. Stains: CD20 (white), CD3 (purple), CD127 (green), ST2 (red), and DAPI (blue). Scale bar: 50 µm. (D) Median importance of cell‐type abundance in the prediction of abundances of other cell types within a spot. (E) Spatial distribution of ILC2 and B‐CD40‐GC‐like in CRC samples by spatial transcriptomic sequencing. (F) Representative flow cytometry plots showing the relative expression of CD38 and CD138 in CD19^+^ B cells under naïve B cells without or with co‐culture with ILC2s. *N* = 3 biological replicates per condition per experiment; results are representative of three independent experiments. Data are presented as mean ± SEM. (G) Bar graphs showing the frequencies of CD38^+^ and CD138^+^ cells within CD19^+^ B cells cultured alone (B cells) or co‐cultured with ILC2s. *N* = 3 biological replicates per group per experiment; data are representative of three independent experiments. Bars indicate mean ± SEM. Statistical significance was determined by a paired two‐tailed Student's *t*‐test. * *p* < .05, ** *p* < .01, *** *p* < .001.

ILC2s have been reported to contribute to the formation and maturation of TLS in pancreatic ductal adenocarcinoma (PDAC).^73^ This finding led us to explore a potential analogous role for ILC2s in CRC. TLS are immune cell aggregates with distinct architectural features, formed in non‐lymphoid tissues under pathological conditions. They are typically characterised by the expression of specific chemokines and the presence of resident immune cells, including activated T cells, B cells, dendritic cells, and myeloid populations.^80^, ^81^ To identify TLS within tumour tissues, we applied three non‐overlapping transcriptional signatures capturing chemokine expression,^81^ immune cell composition,^82^ and additional immunomodulatory factors.^80^ Notably, the ILC2 gene signature exhibited a strong correlation with all three TLS signatures (Figure 5F). To further validate the spatial relationship between ILC2s and TLS in CRC, we analysed two previously published spatial transcriptomics datasets containing TLS regions. Firstly, using the SPECAT package, we annotated spatial transcriptomics spots into tumour, stromal, and tumour–stroma interface regions. The TLS‐associated gene signature was then applied to identify TLS regions, which were found to be predominantly localised within tumour areas. By employing cell2location method, we found ILC2s were localised within TLS regions (Figures 6A and S6A). This was further validated by IF, showing clear accumulation of ILC2 (CD127^+^ST2^+^CD3^−^CD20^−^) within TLS (Figure 6B), with nearly no presence in non‐TLS regions (Figure 6C), aligning with the transcriptomics analysis. Given the well‐known role of TLS in anti‐tumour immunity, we examined PDAC and gastric cancer (GC) spatial transcriptomics and observed similar ILC2s localisation within TLS (Figure S7A), indicating that ILC2 enrichment within TLS may represent a conserved feature across various cancer types

We further found that ILC2s were in closest proximity to B‐CD40‐like cells in TLS using mistyR analysis (Figure 6D and E), which was subsequently validated by IF stainging(Figure S6B). The close spatial association between ILC2s and B cells within TLS raised the question of whether ILC2s could functionally influence B cell maturation. To address this, we performed in vitro co‐culture experiments using purified ILC2s and naïve B cells. We found that B cells co‐cultured with ILC2s displayed substantially elevated expression of CD138 and CD38 compared to B cells cultured alone (Figure 6F). Collectively, these findings suggest that ILC2s may contribute to the functional maturation of TLS‐resident B cells, and could support TLS‐mediated antitumour immunity in CRC. Further experimental validation is required to definitively establish these causal relationships.

### 3.6 Baseline levels of intratumoural ILC2s are associated with patient outcomes and immunotherapy response

**FIGURE 7:**
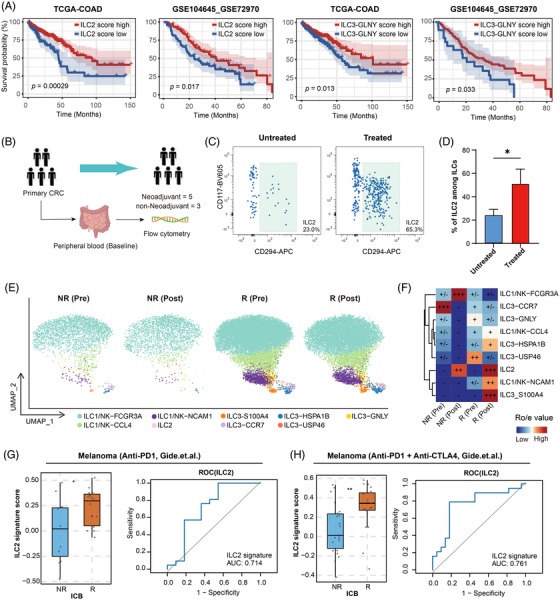
Baseline levels of intratumoural ILC2s are associated with patient outcomes and immunotherapy response. (A) Kaplan–Meier overall survival curves for ILC2 and ILC3‐GNLY signatures in CRC cohorts. Left: ILC2 signature in TCGA datasets and GEO cohorts. Right: ILC3‐GNLY signature in TCGA datasets and GEO cohorts. (B) Schematic of CRC patients treated with ICB therapy used as discovery cohort. (C) Flow cytometry analysis of ILC2 in peripheral blood from patients without or with ICB. (D) The ratio of ILC2 quantified in peripheral blood from patients without or with ICB treatment (*n* = 8). Statistical significance was determined using a two‐sided paired *t*‐test. (E) UMAP plots and proportions of ILC2 clusters in pre‐treatment and post‐treatment tissues from patients with different responses in a CRC cohort (*n* = 22). R: responders; NR: non‐responders. (F) Tissue preference of each cluster estimated by the STARTRAC‐dist index. +++, Ro/e > 2; ++, 1.5 < Ro/e ≤ 2; +, 1 ≤ Ro/e ≤ 1.5; +/−, 0.5 < Ro/e < 1; −, Ro/e < 0.5; in which Ro/e denotes the ratio of observed to expected cell number. R: responders; NR: non‐responders. (G) Predictive value of ILC2 for clinical benefits in melanoma patients receiving anti‐PD1 therapy (*n* = 41). Error bars represent the mean ± SEM. Statistical significance was determined using an unpaired two‐sided *t*‐test. R: responders; NR: non‐responders. (I) Predictive value of ILC2 for clinical benefits in melanoma patients receiving anti‐PD1 and anti‐CTLA4 therapy (*n* = 32). Error bars represent the mean ± SEM. Statistical significance was determined using an unpaired two‐sided *t*‐test. R: responders; NR: non‐responders.

Previous analyses have suggested that ILC2s may contribute to antitumour immunity via the TLS axis, and a well‐established correlation exists between TLS presence and improved patient prognosis and therapeutic responsiveness.^33^, ^80^ However, the precise association between ILC2s and patient survival and therapeutic response in CRC remains unclear. In this study, we aim to address these gaps by investigating the role of ILC2s in predicting clinical outcomes and treatment efficacy. We first performed Kaplan–Meier survival analysis using TCGA datasets, which revealed that high expression of ILC2 signature genes was significantly associated with improved disease‐free survival. This was further validated in the independent GEO cohorts, which consistently showed favourable prognostic associations. Similarly, high abundance of ILC3‐GNLY also demonstrated a strong association with improved survival (Figure 7A). In addition, we found a significantly higher proportion of ILC2s in the treated cohort compared to the untreated cohort in our internal cohort of CRC patients (*n* = 8) (Figure 7B–D), suggesting a possible association between therapy and increased ILC2 frequencies.

Given the growing use of ICB in cancer therapy, we further investigated whether ILC2 levels could serve as predictive biomarkers of immunotherapy response. We analysed scRNA‐seq data from pre‐ and post‐treatment tumour biopsies of CRC patients receiving neoadjuvant immunotherapy. After quality control, 32 098 ILCs were retained and classified into nine transcriptionally distinct clusters (Figure 7E). Notably, treatment responders exhibited a significant expansion of ILC2 after treatment, with higher ILC2 levels compared to baseline. While ILC2 levels increased in all patients, responders consistently showed the highest levels (Figure 7F). Collectively, these findings suggest that intratumoural ILC2 abundance is associated with favourable patient outcomes and may serve as a potential predictive biomarker for immunotherapy responsiveness in CRC. Given the expanding role of immunotherapy in the treatment of various malignancies, we further extended our analysis to assess whether ILC2‐related transcriptional signatures could serve as predictive biomarkers across multiple tumour types. In melanoma patients treated with anti‐PD‐1 monotherapy, higher ILC2 signature scores were observed in responders compared to non‐responders (Figure 7G). A similar pattern was detected in melanoma patients receiving combined anti‐PD‐1 and anti‐CTLA4 treatment. Those results support the cross‐tumour applicability of ILC2 signatures as indicators of immunotherapeutic responsiveness (Figure 7H).

Taken together, these findings underscore the potential of ILC2‐related gene signatures as pan‐cancer biomarkers that can inform prognosis and predict immunotherapeutic responsiveness, thereby offering valuable insights for the development of precision oncology strategies.

## 4 DISCUSSION

Extensive studies have highlighted the important role of ILCs in TME,^16^, ^26^ yet the contribution of these cells has not been systematically explored in CRC. To address this gap, we applied scRNA‐seq and spatial transcriptome to delineate the composition, states, and spatial organisation of ILCs and clarify how these properties inform their function in CRC.

Our findings reveal that tissue‐resident ILC3s are not homogeneous but comprise transcriptionally and functionally distinct states that are differentially distributed between normal mucosa and tumour tissue. Whereas mucosal ILC3s are linked to homeostatic regulation, the tumour‐associated ILC3 subsets display stress‐adapted, pro‐inflammatory, or cytotoxic programs, reflecting the inherent plasticity of ILC3s. Most strikingly, we identified an ILC3 subset designated ILC3‐GNLY that expresses genes characteristic of both canonical ILC3 identity and type‐1 immunity, including cytotoxic effector molecules. This is consistent with earlier studies showing that cytokine signalling can reprogram ILC3s towards ILC1‐like states,^29^, ^83^ with the inferred trajectory likely reflecting context‐dependent transcriptional changes shaped by the intestinal tumour environment.^84^ Despite findings consistent with the previous study, lineage tracing analysis was remain necessary to confirm the transdifferentiated process from ILC3 to ILC1.

Among tumour‐infiltrating ILC subsets, ILC2s emerged as a distinct subset, with important spatial and functional characteristics. Nearly absent in normal intestinal mucosa, ILC2s were selectively enriched within tumour tissues and preferentially localised within TLS. Critically, in vitro co‐culture experiments demonstrated that ILC2s promote naïve B cell maturation, as evidenced by elevated expression of CD138 and CD38. While the contribution of ILC3s to TLS development has been a major focus of previous research, our study highlights a parallel and understudied role for ILC2s in this process. Ikeda et al. found that LTi‐like ILC3s promote TLS formation in CRC through lymphotoxin signalling,^79^ while Carrega et al. reported similar ILC3‐mediated TLS organisation in non‐small cell lung cancer.^64^ Our data reveal substantial ILC3 heterogeneity within CRC tumours, raising the question of which ILC3 subsets contribute to TLS formation in this context. Given that both ILC2s and certain ILC3 subsets are associated with TLS in our spatial analyses, these populations may play distinct roles shaped by local microenvironmental cues. However, the precise contributions of each ILC subset to TLS biogenesis, maturation, and function remain to be elucidated through mechanistic studies.

Regarding the prognostic significance of ILC2s in CRC, our data showed that elevated intratumoural ILC2s abundance correlated with TLS gene signatures and improved patient survival. Importantly, ILC2 signatures also predicted immunotherapy responsiveness across multiple cancer cohorts, including CRC and melanoma, suggesting that TLS‐associated ILC2s may serve as biomarkers of organised anti‐tumour immunity. Our findings present a different perspective from prior studies. Prior investigations have linked ILC2‐derived IL‐13 secretion to the promotion of colorectal tumour progression via suppressive immune signalling. Similarly, elevated ILC2 transcriptomic profiles have been identified as a correlate of poor survival outcomes in patients with prostate cancer.^77^, ^78^ Our data suggest a distinct role in the context of TLS biology. We propose that these discrepancies reflect context‐dependent ILC2 functions determined by spatial localisation, disease stage, tumour type, and the pre‐existing immune landscape. Specifically, TLS‐resident ILC2s may support organised adaptive immunity, whereas stromal ILC2s may promote immunosuppressive networks. This spatial dichotomy suggests that ILC2s are neither uniformly pro‐ nor anti‐tumoural, but rather plastic modulators whose function depends on their tissue niche. Besides, whether ILC2s causally contribute to therapeutic responses or merely reflect a favourable immune contexture requires further investigation in preclinical models.

In the future, single‐cell multiome sequencing (Chromatin accessibility, RNA transcription and protein expression) and high‐resolution spatial profiling will be needed to obtain orthogonal molecular and spatial measurements that refine ILC subset delineation. These multimodal datasets will enable direct assessment of protein‐RNA concordance and transcriptional‐epigenetic coupling, thereby resolving microenvironmental cues that imprint distinct ILC states across tissues. This framework will also mitigate annotation challenges inherent to scRNA‐seq analyses, particularly the sporadic detection of low‐level *CD3D*, *CD3E*, or *CD4* transcripts in human ILC1 subsets,^85^ which can obscure lineage boundaries between T cells and ILCs. Mechanistically, inducible fate‐mapping strategies combined with syngeneic and patient‐derived tumour models will allow us to trace ILC development with temporal precision and test whether inferred state transitions occur in vivo under immune pressure. CRISPR‐based perturbation of key transcriptional ‘hub’ genes will further elucidate the molecular switches governing ILC differentiation and functional adaptation.

## 5 CONCLUSIONS

This study provides insights into the cellular origin and functional roles of intestinal ILC subsets in CRC through integrated multi‐omics analysis. We identified tissue‐compartment‐associated distribution patterns of ILC subsets: ILC3‐CD83 cells are predominantly tissue‐resident in the intestine with transcriptional features resembling fetal gut ILC3s, while ILC2 and ILC3‐S100A4 cells are more prevalent in bone marrow and peripheral blood, with transcriptional profiles that differ across anatomical sites. Additionally, we observed substantial transcriptional heterogeneity among ILC3 subsets. Notably, a subset designated ILC3‐GNLY exhibited dual features, expressing both ILC3‐associated markers and genes characteristic of type‐1 immunity. Furthermore, ILC2s were found to be enriched within tertiary lymphoid structures in tumour tissues. *In* *vitro* co‐culture experiments demonstrated that ILC2s can promote B cell maturation. High ILC2 signature expression was associated with improved prognosis and favourable response to immunotherapy across multiple cohorts.

## AUTHOR CONTRIBUTIONS

Y.G., B.L., C.Z. and C. Liu (Chen) conceived and supervised this study; S.Z. and Q.F. designed and prepared the figures; Z.X., Q.F. and X.Y. performed immunofluorescence assay; Q.F., X.S. and C. Liu (Chen) performed flow cytometry analysis and sorting; X.S. and C. Liu (Chen) prepared singlecell library for scRNA‐seq; S.Z., G.Y. and G.Y. performed bioinformatics analysis. S.Z., Q.F., Z.X., G.Y., X.S., X.Y., S.W., C. Liu (Chao) and C. Liu (Chen) wrote the manuscript.

## ETHICS STATEMENT

All experiments were performed in accordance with protocols approved by the Ethics Committee of Chinese PLA General Hospital (approval number: S2019‐177‐01 and S2024‐523‐01), and the regulations of the Declaration of Helsinki. Written informed consent was obtained from all participants prior to their involvement in the study.

## CONFLICT OF INTEREST STATEMENT

The authors declare there is no conflict of interest regarding the publication of this paper.

## CONSENT FOR PUBLICATION

Written informed consent was also obtained from each patient for publication of their data.

## Supporting information



Supporting Information

Supporting Information

## Data Availability

We downloaded single‐cell RNA‐sequencing datasets from HRA (HRA000919^60^) and GEO (GSE236581,^61^ GSE178341^62^ GSE163587^86^) and integrated them with our unpublished data, yielding a combined cohort of 42 donors for downstream analyses. These datasets include peripheral blood, adjacent normal intestinal mucosa collected under non‐stimulated baseline conditions, and primary colorectal cancer (CRC) tissues from both healthy individuals and patients. Specifically, HRA000919 was generated to examine the heterogeneity of human ILCs by scRNA‐seq in normal intestinal mucosa, CRC tissue, and peripheral blood obtained from healthy donors and CRC patients. GSE236581 contains data from CRC patients, with treatment information. GSE178341 includes primary treatment‐naïve CRC tissues and matched adjacent tumour samples, while GSE163587 consists of ILCs in early human fetal intestinal tissues (8‐12 PCW). In addition, spatial transcriptomics data for CRC tissues can be accessed through the Cancer Diversity database, and data for normal intestinal mucosa are available from 10× Genomics. Immunotherapy cohort data for melanoma (Gide cohort) are available from the Tumour Immune Dysfunction and Exclusion database. In addition, bulk RNA‐seq data of sort‐purified human ILC3s from colon surgical‐resection samples of patients with CRC were obtained from GSE165814^63^ Moreover, publicly available bulk RNA sequencing datasets, including gene expression matrices for CRC patients treated with FOLFOX or FOLFIRI, were obtained from the Springer Nature Figshare repository. Transcriptomic profiles and matched clinical metadata for the TCGA‐COAD and TCGA‐READ cohorts were acquired via the UCSC Xena browser. Detailed clinical metadata for these donors are available in the original publications associated with each dataset. In addition, an in‐house dataset was generated from flow cytometry‐sorted ILCs isolated from the bone marrow of two healthy donors. Raw data are accessible by contacting the corresponding author.
