# Supplementary material for: Decoding innate lymphoid cell heterogeneity and plasticity in colorectal cancer
Source: Clin Transl Med. 2026 Jan 13;16(1):e70593. doi: 10.1002/ctm2.70593 (PMC12796840; doi:10.1002/ctm2.70593)
Supplement: Supplementary file 1 — Supporting Information [file CTM2-16-e70593-s001.docx]

Table S1. Key resources table

| Antibodies | SOURCE | IDENTIFIER |
| --- | --- | --- |
| Anti-human CD45 BV42 | Biolegend | Cat# 304032; RRID: AB_2561357 |
| Anti-human CD11c FITC | Biolegend | Cat# 337214; RRID: AB_2129792 |
| Anti-human CD14 FITC | Biolegend | Cat# 301804; RRID: AB_314186 |
| Anti-human CD19 FITC | Biolegend | Cat# 302206; RRID: AB_314236 |
| Anti-human CD34 FITC | BD | Cat# 555821; RRID: AB_396150 |
| Anti-human CD94 FITC | Biolegend | Cat# 305504; RRID: AB_314534 |
| Anti-human FcεRIa FITC | Biolegend | Cat# 334608; RRID: AB_1227653 |
| Anti-human CD3 APC/Cyanine7 | Biolegend | Cat# 300426; RRID: AB_830755 |
| Anti-human CD127 PE/Cyanine7 | eBioscience | Cat# 25-1278-42; RRID: AB_1659672 |
| Anti-human CD161 PE | Biolegend | Cat# 339904; RRID: AB_1501083 |
| Anti-human CD117 BV605 | Biolegend | Cat# 313218; RRID: AB_2562025 |
| Anti-human CRTH2 APC | BD | Cat# 566785; RRID: AB_2869865 |
| 7-AAD Viability Staining Solution | eBioscience | Cat# 00-6993-50 |
| ST2 Polyclonal Antibody | Abcam | Cat# ab25877; RRID: AB_448870 |
| Recombinant Anti-human CD3 antibody [SP162] | Abcam | Cat# ab135372; RRID: AB_2884903 |
| Recombinant Anti-human CD20 antibody [SP32] | Abcam | Cat# ab64088; RRID: AB_1139386 |
| CD127 Polyclonal Antibody | Invitrogen | Cat# PA5-97870; RRID: AB_2812484 |
| [Recombinant Anti-Hsp70 antibody](https://rrid.site/data/record/nif-0000-07730-1/AB_2910093/resolver?q=ab181606&l=ab181606&i=rrid:ab_2910093-2845549) | Abcam | Cat# ab181606, RRID:AB_2910093 |
| Donkey anti-rabbitlgGBV421 | Biolegend | Cat# 406410, RRID:AB_10897810 |
| Anti-human CD45 FITC | BD | Cat# 555482, RRID:AB_395874 |
| Anti-human CD11c APC/Cyanine7 | Biolegend | Cat# 337217, RRID:AB_10661724 |
| Anti-human CD14 APC/Cyanine7 | Biolegend | Cat# 325620, RRID:AB_830693 |
| Anti-human CD19 APC/Cyanine7 | BD | Cat# 557791, RRID:AB_396873 |
| Anti-human CD34 APC/Cyanine7 | Biolegend | Cat# 343514, RRID:AB_1877168 |
| Anti-human FcεRIa APC/Cyanine7 | Biolegend | Cat# 334632, RRID:AB_2571904 |
| Anti-human CD161 PE/Cyanine7 | Biolegend | Cat# 339917, RRID:AB_1112616 |
| Anti-human SELL BV786 | BD | Cat# 565311, RRID:AB_2744439 |
| Anti-human CD69 PE | Biolegend | Cat# 310905, RRID:AB_314840 |
| Anti-human CD94 PerCP | BD | Cat# 562361, RRID:AB_11152081 |
| Anti-human CD45 PerCP | eBioscience | Cat# 45-0459-42, RRID:AB_10717530 |
| Anti-human IFN-γ PE | Biolegend | Cat# 502508, RRID:AB_315233 |
| Anti-human c-FOS AF488 | Biolegend | Cat# 600753, RRID:AB_3097468 |
| Anti-human IL-17A PE | Biolegend | Cat# 512305, RRID:AB_961395 |
| Anti-human CD19 BV605 | BD | Cat# 562654, RRID:AB_2909453 |

| REAGENT | SOURCE | IDENTIFIER |
| --- | --- | --- |
| Recombinant Human IL-2 | PeproTECH | 200-02 |
| Recombinant Human IL-4 | PeproTECH | 200-04 |
| Recombinant Human IL-7 | PeproTECH | 200-07 |
| Recombinant Human IL-21 | PeproTECH | 200-21 |
| Ficoll-Paque | Cytiva | 17144003 |
| RPMI-1640 | Gibco | C118755008T |
| Fetal bovine serum | Gibco | A5256701 |
| Collagenase I | Sigma-Aldrich | C2674 |
| Collagenase IV | Gibco | 17104019 |
| DNase I | Roche | [10104159001](https://www.sigmaaldrich.cn/CN/zh/product/roche/10104159001) |
| Zombie Aqua™ Fixable Viability Kit | Biolegend | 423101 |
| EasySep™ Human Naïve B Cell Isolation Kit | STEMCELL | 17254 |
| Human Lineage Cell Depletion Kit | Miltenyi Biotec | 130-092-211 |
| human AB serum | Sigma-Aldrich | H4522 |
| penicillin–streptomycin | Gibco | 15140122 |
| L-glutamine | Gibco | 25030081 |
| sodium pyruvate | Gibco | 11360070 |
| HEPES | Gibco | 15630106 |
| MEM non-essential amino acids | Gibco | 11140050 |
| 2-mercaptoethanol | Gibco | 31350010 |
| Human TruStain FcX™ | Biolegend | 422302 |
| Cell Staining Buffer | Biolegend | 420201 |
| Foxp3 / Transcription Factor Staining Buffer Set | eBioscience | 00-5523-00 |
