# Supplementary figures and images for: Decoding innate lymphoid cell heterogeneity and plasticity in colorectal cancer
Source: Clin Transl Med. 2026 Jan 13;16(1):e70593. doi: 10.1002/ctm2.70593 (PMC12796840; doi:10.1002/ctm2.70593)

**FigS1**

**A**

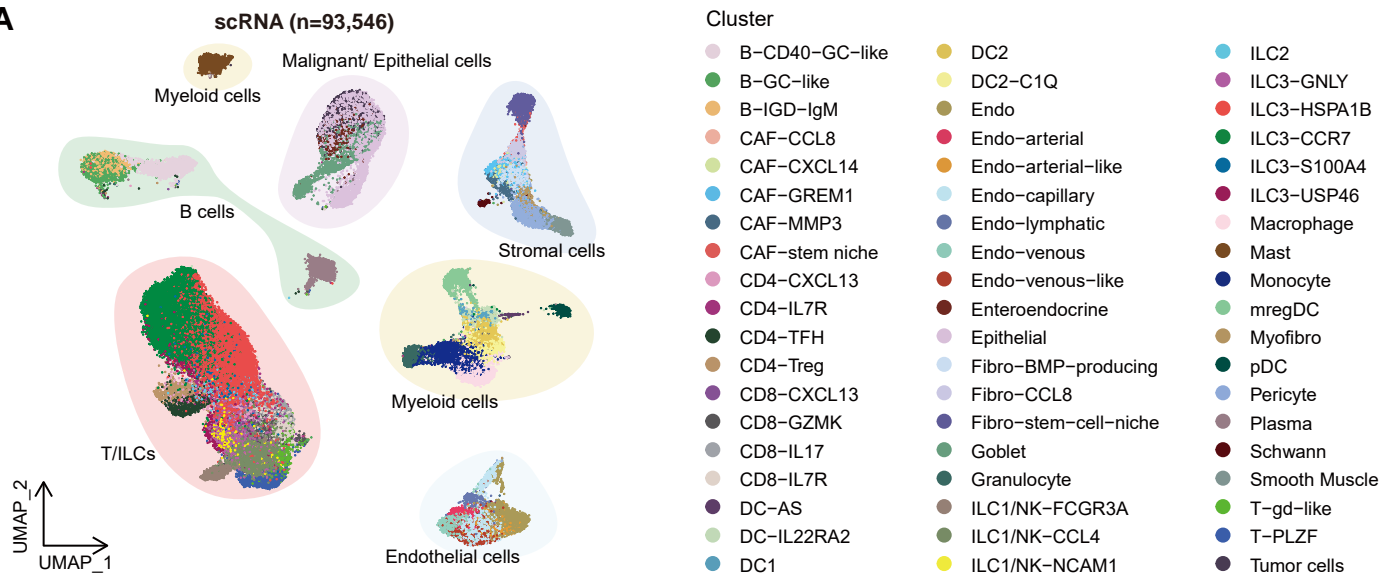

**B**

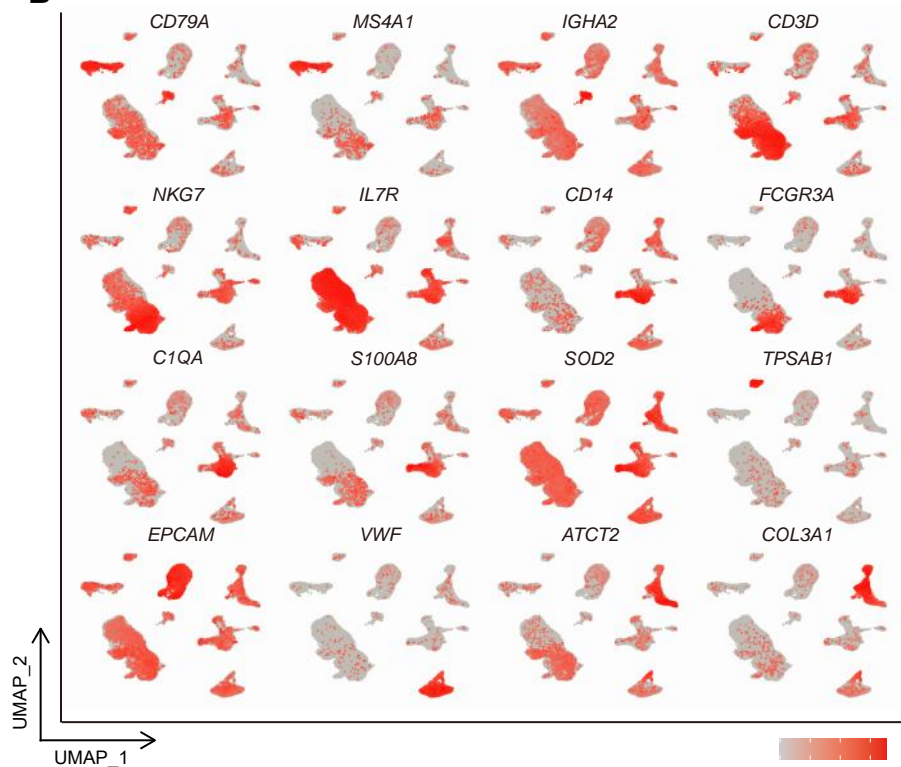

**C**

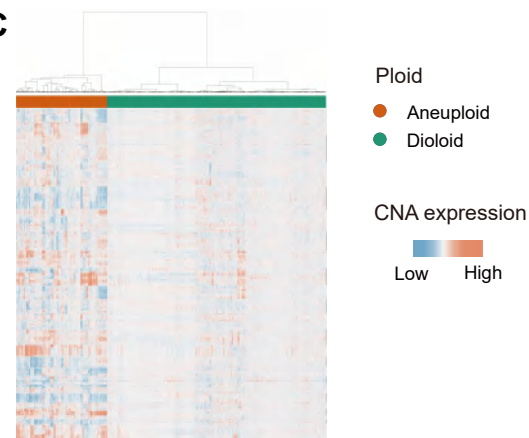

**D**

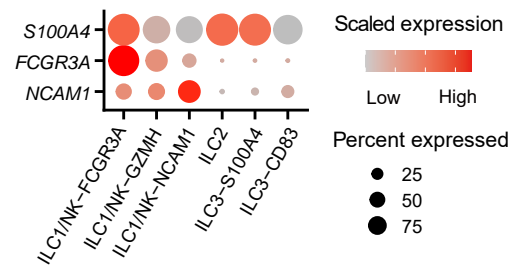

**E**

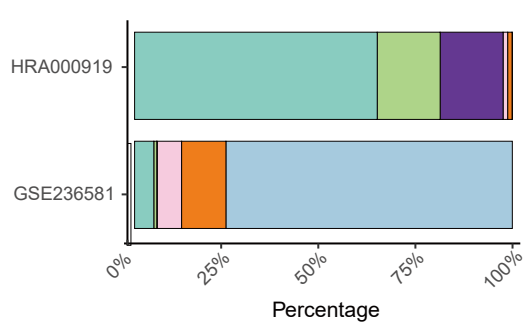

**G**

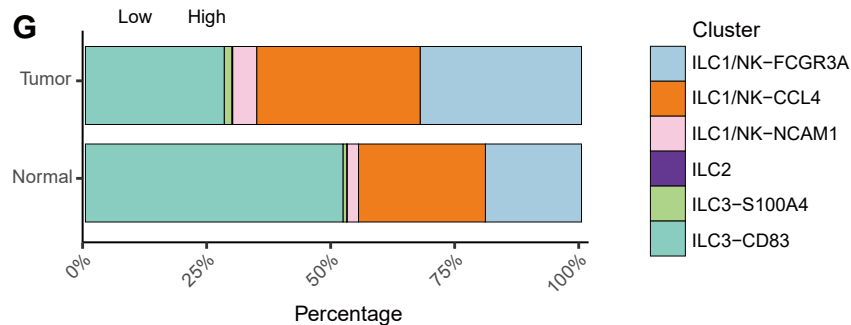

**F**

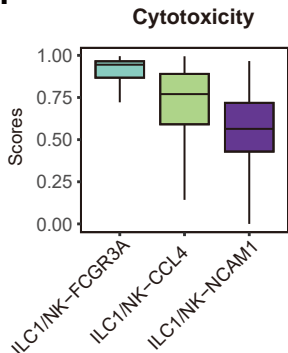

**H**

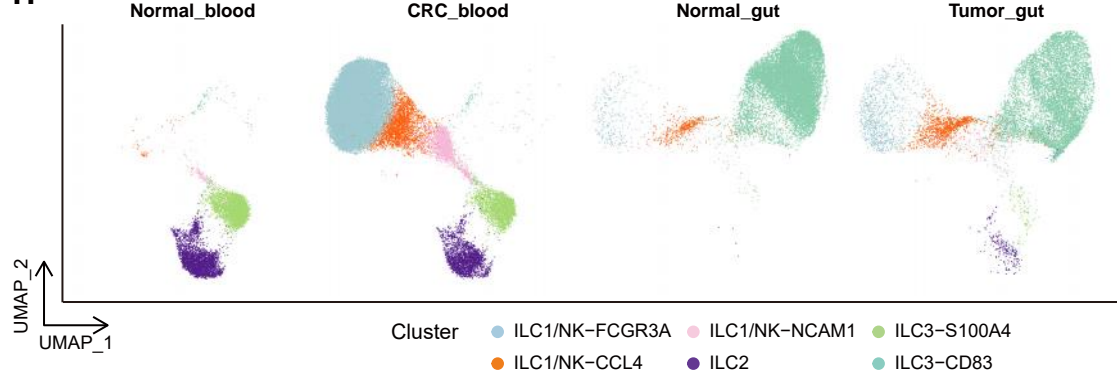

FigS2

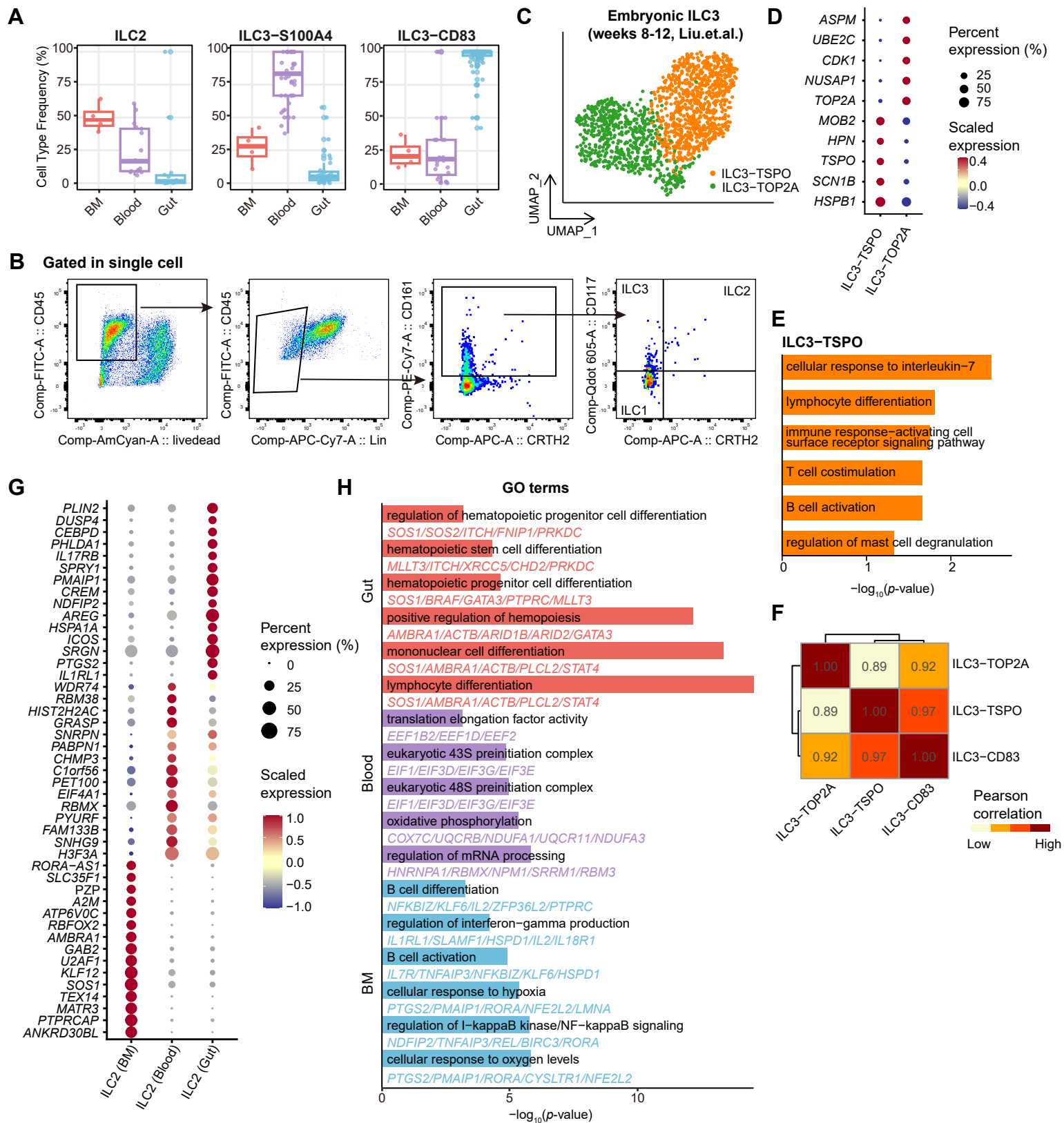

**A**

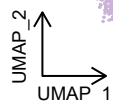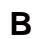

Scaled expression

FigS4

A

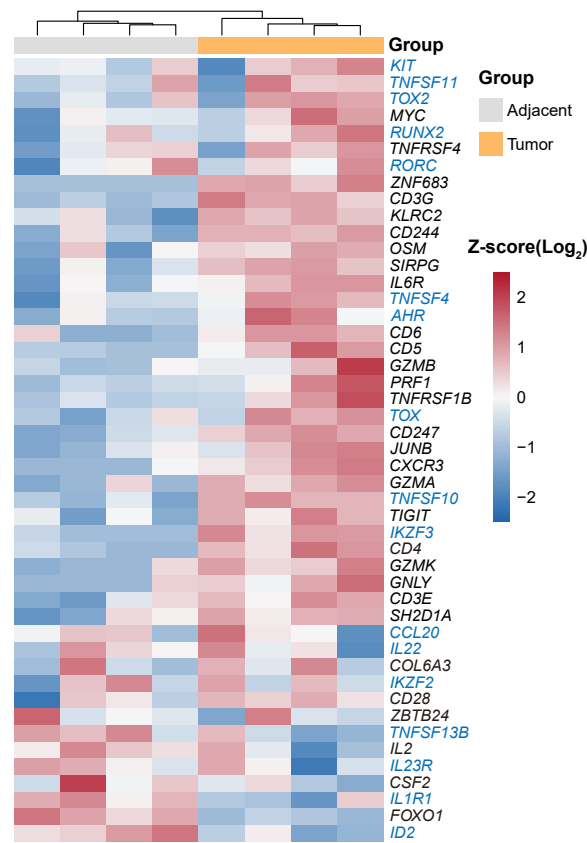

B

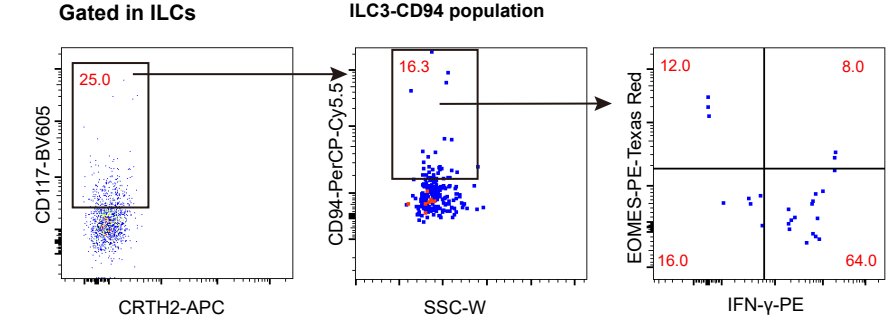

C

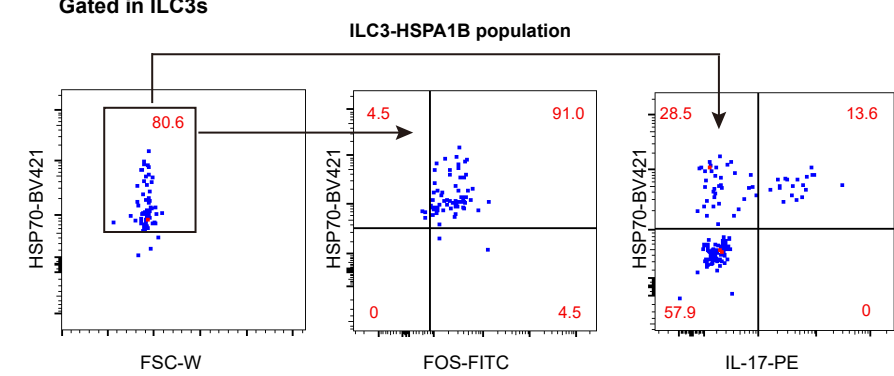

FigS5

A

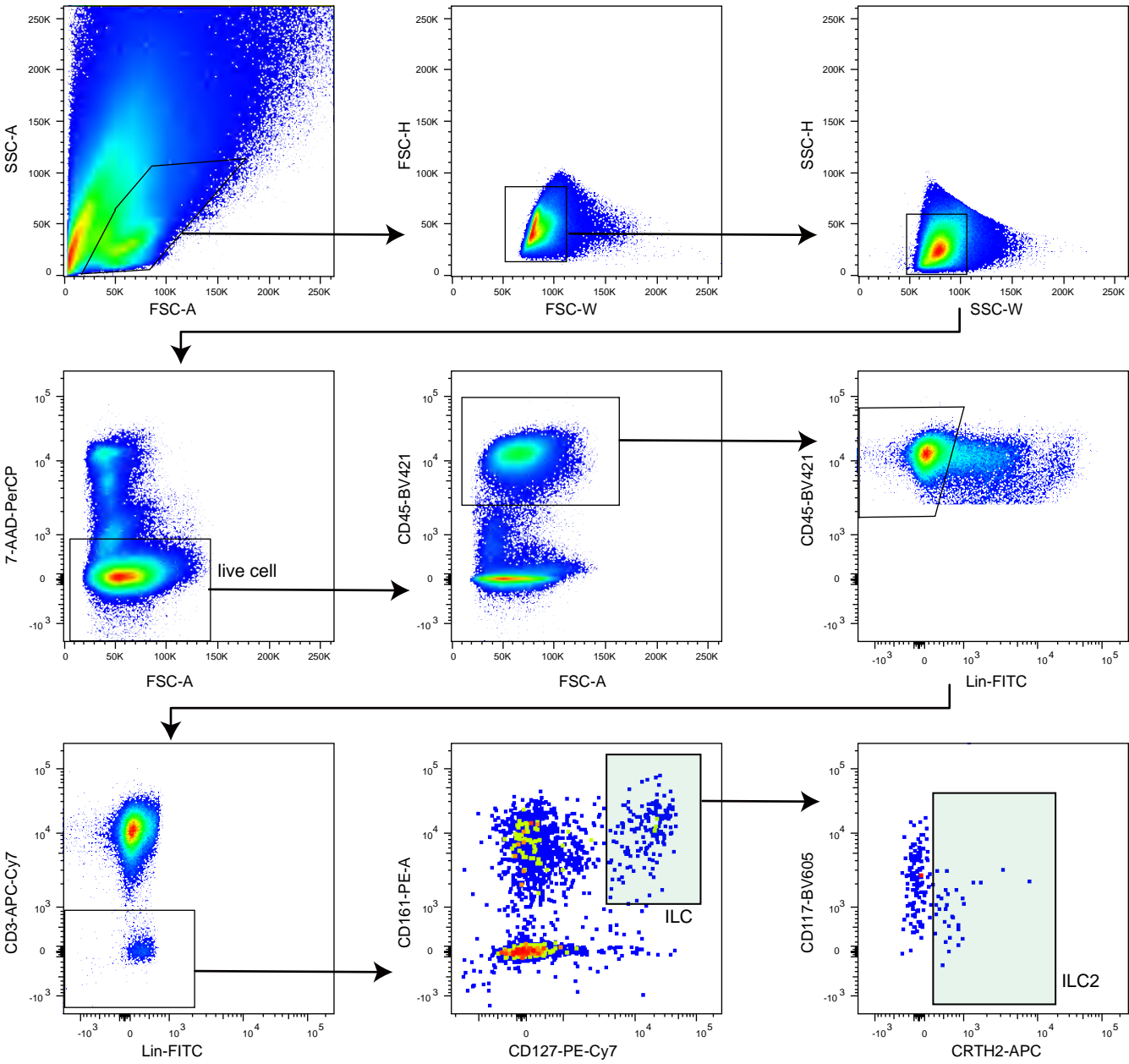

FigS6

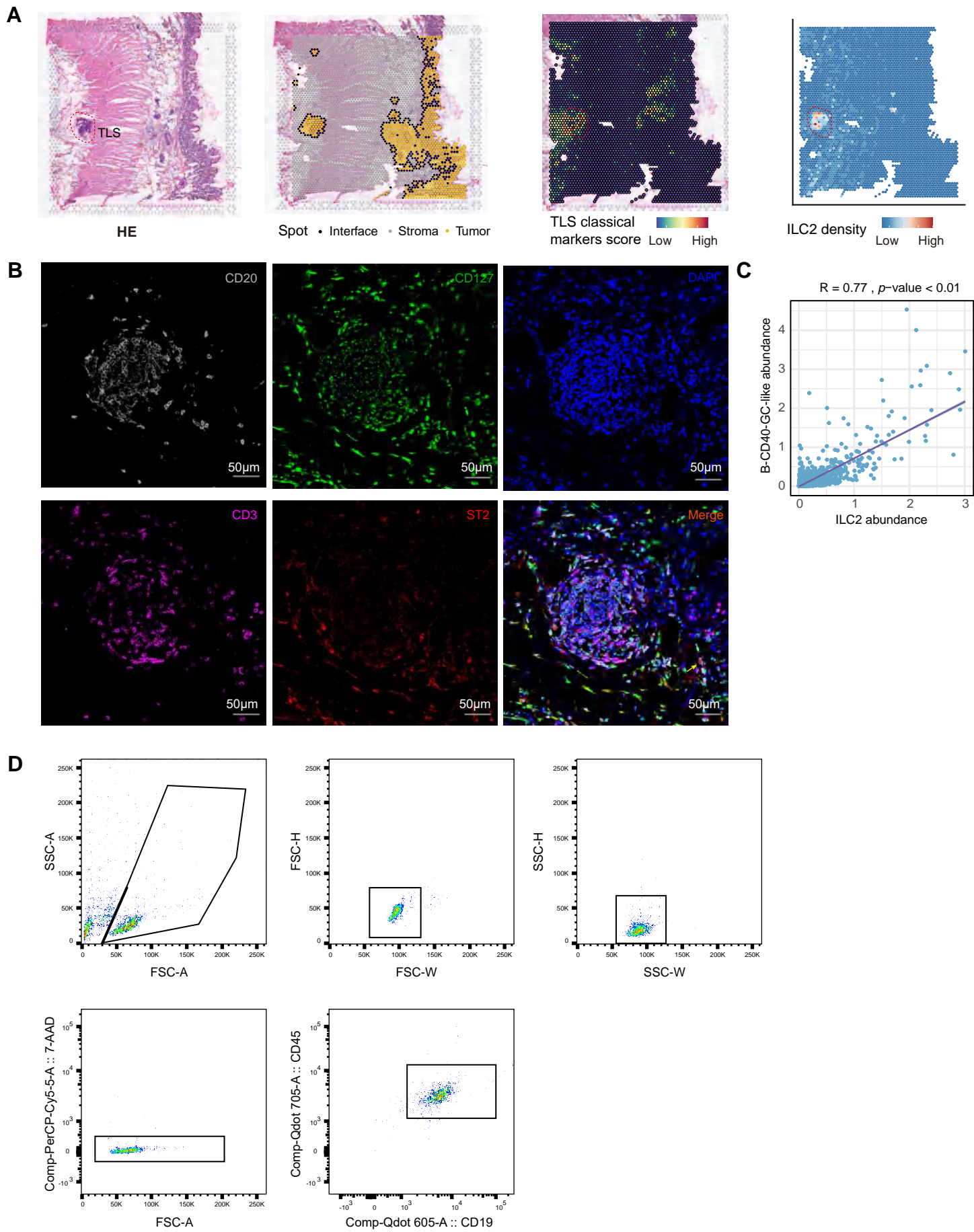

FigS7

A

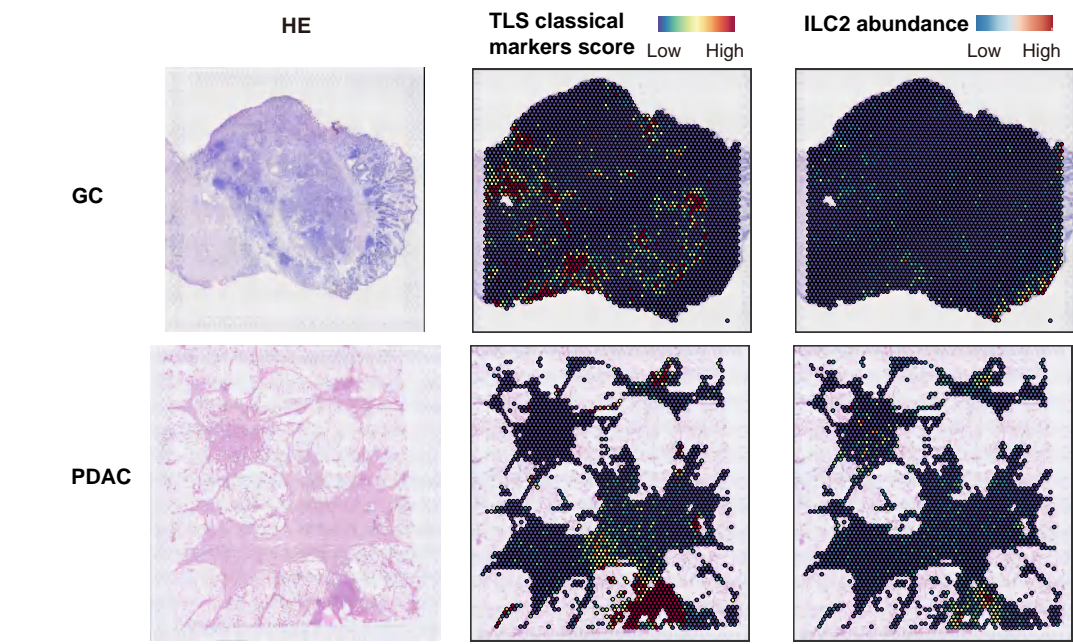

Supplement: Supplementary file 2 — Supporting Information [file CTM2-16-e70593-s002.pdf]
